# Germination speed modulates priority effects: Evidence from a large‐scale field study

**DOI:** 10.1002/ecy.70291

**Published:** 2026-01-23

**Authors:** Tamara L. H. van Steijn, Paul Kardol, Roland Jansson, Judith M. Sarneel

**Affiliations:** ^1^ Department of Ecology and Environmental Science Umeå University Umeå Sweden; ^2^ Department of Forest Ecology and Management Swedish University of Agricultural Sciences Umeå Sweden; ^3^ Department of Forest Mycology and Plant Swedish University of Agricultural Sciences Uppsala Sweden

**Keywords:** boreal, germination speed, phenology, plant community assembly, plant competition, priority effects, seed density, temperate

## Abstract

Priority effects, where species that arrive first influence later arriving species, are often considered in terms of seed arrival time. However, the timing of seedling emergence may play a more critical role, as this defines when plants start interacting. Further, initial seed density may also be important, allowing early‐arriving species with low initial seed density to overcome seed limitation, while also potentially allowing late‐arriving high‐density species to overcome the disadvantage of arriving late. In this large‐scale, multi‐site field experiment, we manipulated species arrival and emergence timing by sowing fast‐ and slow‐germinating meadow species in various arrival orders and seed densities across two climatically contrasting sites in Sweden. Our findings demonstrate that germination speed modulates the strength and direction of priority effects: fast‐germinating species were less affected by both early‐ and late arrival. Conversely, slow‐germinating species were disadvantaged by late arrival and benefited significantly from early arrival, particularly at the more productive, northern site with shorter growing seasons. Contrary to expectations, initial sowing density had limited and inconsistent effects on priority effect outcomes. These results highlight that emergence timing, not just seed arrival, is a key aspect of priority effects, influencing plant competition and community structure. Furthermore, the context dependency across sites emphasizes the importance of environmental conditions in modulating priority effects, with implications for predicting vegetation dynamics under climate change.

## INTRODUCTION

Species often arrive sequentially at a site due to variations in the timing of phenological events such as seed dispersal (Heydel & Tackenberg, 2017; Sarneel et al., 2016). Within the local species pool, phenological differences can cause species to establish in varying arrival orders when communities reassemble after a disturbance (Fukami, 2015). At broader spatial scales, differences in arrival order can also arise from processes such as range expansion and biological invasion (Hess et al., 2019).

Species that arrive later often experience different interactions with earlier arriving species compared to if they had arrived simultaneously, a phenomenon known as priority effects. These effects arise through preemption of resources (i.e., niche preemption) or modification of the environment (i.e., niche modification, Fukami, 2015). Longer intervals between species arrivals have been shown to strengthen priority effects by giving early‐arriving species more time to preempt resources or modify their environment (Kardol et al., 2013). This, in turn, can alter competitive hierarchies, potentially leading to long‐term shifts in community composition (van Steijn et al., 2025).

In addition to differences in the arrival time of *seeds*, differences in the timing of emergence can influence the arrival time of *seedlings*. This is because of phenological differences between species, ranging from species that germinate early and fast in the seasons to slower, bet‐hedging species that spread germination throughout the season (Tielbörger et al., 2012; Verdú & Traveset, 2005). Since plants typically cannot start niche preemption or modification until they have germinated, the arrival interval between seedlings, rather than seeds, may be much more relevant when studying priority effects. The timing of seedling emergence is already recognized as an important factor in establishing competitive hierarchies among species (Ross & Harper, 1972), and early emergence is considered a common strategy among invasive plant species, allowing them to outcompete native species (Cleland et al., 2015; Wainwright et al., 2012). This shows that species that germinate faster might cause priority effects on species that germinate slower, even when they have arrived at the same time or have been present in the seed bank together, if early emergence allows species to preempt resources or modify the environment (Blackford et al., 2020).

Zou and Rudolf (2023) point out that priority effects can be trait‐dependent, such as phenological differences as described above, but they can also be frequency‐dependent. This type of priority effect arises when species gain a head start that lasts for multiple generations, leading to higher population densities compared to late‐arriving species. However, frequency‐dependent priority effects are not often tested in plant communities because the arrival interval is usually kept shorter than the lifespan of the experimental species, and further because initial densities are kept constant among species (von Gillhaussen et al., 2014). Species with low initial densities may be particularly sensitive to arrival order. Specifically, arriving not only late but also in low densities may compound to reduce a species' ability to compete with established plants. In contrast, early arrival reduces both intra‐ and interspecific competition, allowing for resource preemption, which may promote rapid growth and confer a competitive advantage over later arrivals (Freckleton & Watkinson, 2001).

Abiotic factors also interact with the strength of priority effects. For example, priority effects are expected to be stronger in more productive environments, since higher nutrient levels promote rapid growth of early‐arriving species, resulting in stronger asymmetric competition (Chase, 2003). However, higher nutrient availability can also create more viable niches, potentially weakening or nullifying priority effects for species capable of filling those niches (Chase, 2003). Further, priority effects have been speculated to be stronger in climates with short growing seasons, where species must quickly secure enough resources to survive impending drought or cold period (Hausmann & Hawkes, 2010). These interactions between species and their environments mean that priority effects can play out differently depending on the abiotic context (Young et al., 2015). This can occur not only between sites, but also within the same site due to spatial heterogeneity. Therefore, conducting experiments across multiple sites and using large plots is crucial for robustly testing priority effects, and could substantially advance our understanding of plant community assembly.

In this study, we tested how variation in the timing of emergence influences the strength of priority effects. The timing of emergence was manipulated by selecting species that differed in their germination speed after sowing, which we defined as the amount of time needed for 50% of seeds to germinate. We calculated germination speeds of 20 common meadow species under standardized, controlled conditions and then classified them as either slow‐ or fast‐germinating. These groups were then further divided to be sown at either high or low density using a split plot design. At two sites with contrasting climates, we established large experimental plots where we introduced the fast‐germinating and slow‐germinating groups either sequentially or simultaneously.

**FIGURE 1:**
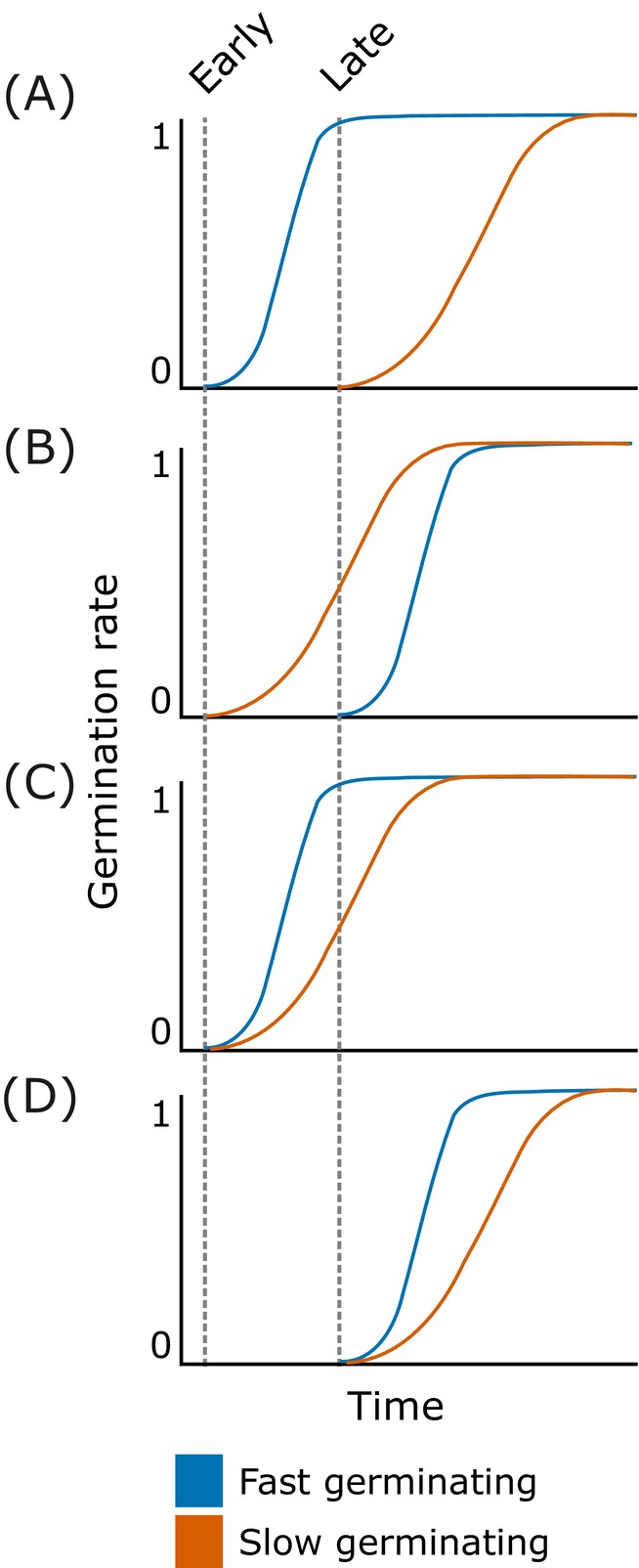
Hypothesized scenarios illustrating how differences in germination speed can influence or create an arrival interval among seedlings. Dashed lines indicate the arrival times of seeds of either a fast‐ or slow‐germinating species. We hypothesize that priority effects should be strongest when the fast‐germinating species arrives first, since this results in the longest interval between emergence (A) and weaker when slow‐germinating species arrive early (B). Further, the small difference in seedling arrival time between fast‐ and slow‐germinating species may even lead to priority effects when they arrive simultaneously (C, D).

**FIGURE 2:**
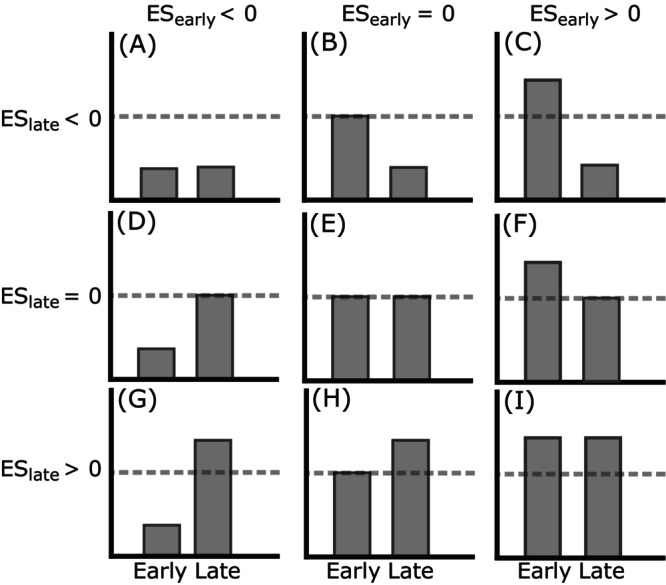
Sequential arrival (indicated by arriving early or late on the *x*‐axis) can lead to various outcomes when compared to simultaneous arrival. This can be for any performance metric, for example, percentage cover, plant biomass, or seed production. There can be no effect, here indicated as effect sizes (ES) that are no different from the dashed line (E). There can be an effect on the early‐arriving species, but not the late‐arriving species (performing worse [D] or better [F]). There can be an effect on the late‐arriving species, but not on the early‐arriving species (performing worse [B] or better [H]). Finally, there can be effects on both species (groups) involved, in any combination of performing, worse or better (A, C, G, I).

We tested the following hypotheses: (1) Sowing the fast‐germinating group before the slow‐germinating group will result in a larger seedling arrival interval (Figure 1A), leading to stronger priority effects. Conversely, sowing the slow‐germinating group before the fast‐germinating group will result in a smaller seedling arrival interval (Figure 1B) and weaker priority effects. These priority effects may manifest as a benefit for early‐arriving species, a disadvantage for late‐arriving species, or both (Figure 2B,C,F). (2) Species sown at low initial densities will experience both a greater disadvantage from late arrival and a greater benefit from early arrival compared to species sown at high initial densities. (3) If our first hypothesis holds, we expect that when both groups are sown together, the fast‐germinating group will outperform the slow‐germinating group and become dominant. This is likely because the small head start provided by faster germination (Figure 1C,D) enables this group to preempt resources more effectively.

## METHODS

### Species selection and grouping

#### Germination speed

First, we purchased seeds of 33 native meadow species native to Sweden, including 19 species that are indicative of high‐nature‐value meadow and pasture land (Jordbruksverket, 2016) and 14 common species found in large areas of Sweden (SLU Artdatabanken 2025, Table 1 and Appendix S1: Table S1).

**TABLE 1:** Plant species used in the experiment organized by germination speed group, functional group, and sowing density with their seed vendor.

Species	Functional group	Group	Sowing density
*Agrostis capillaris* (L.)^a^	Grass	Fast	High
*Festuca rubra* (L.)^a^	Grass	Fast	High
*Deschampsia cespitosa* (L.)^b^	Grass	Fast	Low
*Poa alpina* (L.)^a^	Grass	Fast	Low
*Galium verum* (L.)^a^	Forb	Fast	High
*Leucanthemum vulgare* (Lam.)^b^	Forb	Fast	High
*Plantago media* (L.)^b^	Forb	Fast	High
*Cardamine pratensis* (L.)^b^	Forb	Fast	Low
*Dianthus deltoides* (L.)^b^	Forb	Fast	Low
*Sedum acre* (L.)^b^	Forb	Fast	Low
*Phalaris arundinacea* (L.)^b^	Grass	Slow	High
*Poa pratensis* (L.)^a^	Grass	Slow	High
*Briza media* (L.)^b^	Grass	Slow	Low
*Calamagrostis epigejos* (L.)^b^	Grass	Slow	Low
*Antennaria dioica* (L. Gaertn.)^b^	Forb	Slow	High
*Campanula persicifolia* (L.)^b^	Forb	Slow	High
*Filipendula vulgaris* (L.)^a^	Forb	Slow	High
*Hypochaeris maculata* (L.)^a^	Forb	Slow	Low
*Pulsatilla vulgaris* (L.)^b^	Forb	Slow	Low
*Succisa pratensis* (L.)^b^	Forb	Slow	Low

^a^ Cruydthoeck (Nijeberkoop, NL).

^b^ Jelitto (Schwarmstedt, DE).

We then determined the germination rate of these species by placing 15 or 30 seeds (depending on availability) on agar plates (pure agar without nutrients, Sigma, Steinheim, Germany), which were sealed with Parafilm to prevent moisture loss. The seeds were incubated in climate chambers at three temperatures: 10°C, 15°C and 20°C, with two or four replicates per temperature, depending on seed availability. We counted and removed germinated seeds every 3 days over an 8‐week period. A seed was considered germinated when its radicle was visible to the naked eye. The germination rate was calculated as the total number of seeds germinated on a plate over the 8‐week period, divided by the total number of seeds sown on that plate. Species with an average germination rate below 20% (mean across all temperatures) were excluded from further analysis.

Next, we used time‐to‐event models (*drc* package in *R*, Ritz et al., 2015; R Core Team, 2023) to calculate the germination speed of each species, defined as the number of days needed for 50% of the seeds to germinate (T50, aligning Onofri et al. (2019)). Note that 100% germination here refers to the amount of seeds that germinated by the end of the 8‐week period, not the amount of seeds that were sown. Time‐to‐event models account for uncertainty caused by “censoring,” where exact germination times are unknown, but the time interval in which germination happened is recorded. Based on the calculated germination speeds, we created a group of 10 fast‐germinating species and a group of 10 slow‐germinating species (four grasses and six forbs for each group). For this, we excluded data from 20°C trials and focused on the 10°C and 15°C trials because germination in early spring is more likely to occur under these lower temperatures. First, we identified the median of all germination speeds (T50), which was 243 h. We then selected our groups based on whether species germination speeds (T50) were higher or lower than the median. For some species, T50 was higher than the median at 10°C and lower than the median at 15°C. In these cases, we prioritized the T50 at 10°C, as earlier germination at lower temperatures is more relevant to our experiment (see Appendix S1: Section S1.2 for raw data and germination curves of individual species).

#### Initial density

To test whether initial species’ densities influence priority effects, we used a split‐plot design where half of the species in each germination‐speed group were sown at high initial densities and the other half at low initial densities. For this, each germination‐speed group, consisting of six forbs and four grasses, was further divided into two groups of three forbs and two grasses. We based the density selection as much as possible on natural abundance in the vegetation based on red list status and vegetation descriptions. However, we assigned certain species solely in order to create a balanced distribution of grasses and forbs between the groups (Appendix S1: Table S2). We chose a seeding ratio of 1:9 (low‐density seeds to high‐density seeds), based on species rank abundance curves, which typically show an exponential decrease in species abundances.

### Experimental sites

#### Selection, history, and preparation

We selected two climatically contrasting sites within the Swedish Infrastructure for Ecosystem Science (SITES, www.fieldsites.se): Lönnstorp in southern Sweden (55°40′04.5″ N, 13°06′35.9″ E) and Röbäcksdalen in northern Sweden (63°48′19.7″ N, 20°14′19.9″ E). Both sites have a history of agricultural use with rotations of various crops. Röbäcksdalen has primarily been used for fodder production, with grass‐clover mixtures rotated with barley, while Lönnstorp has been cultivated with winter wheat, barley, sugar beets, and rapeseed. In preparation for the experiments in 2021, the fields were plowed in the autumn of 2020. Three weeks before the start of the experiment, the experimental sites were treated with Roundup Gold to suppress the growth of agricultural weeds (3 L per hectare) and raked after.

#### Climatic differences and soil properties

We obtained air temperature data from the SITES database (SITESa, 2024; SITESb, 2024) and precipitation data from the Swedish Meteorological and Hydrological Institute (SMHI, 2025).

Soil particle composition data were obtained from the SITES research stations. To compare soil moisture at the start of the experiment, three soil samples were collected from each plot to a depth of 10 cm. After homogenizing the samples within each plot, soil moisture was measured by weighing 15 g of soil before and after drying at 105°C for 48 h to determine the mass loss.

To compare soil nutrient levels, we prepared soil extracts by shaking 5 g of fresh soil with 25 mL of Milli‐Q water, first by hand, then for 3 h with an orbital shaker at 50% speed, before letting extracts settle overnight. After filtering with 0.45 μm Filtropur filters, we determined nitrate–nitrogen (NO3‐N), ammonium–nitrogen (NH4‐N), and phosphate–phosphorus (PO4‐P) concentrations (for methods, see Appendix S1: Section S3.1). In the last year (2024), the soil pH was compared using soil extracts as described above, followed by measuring with a pH meter (Mettler Delta 340, Mettler Toledo, Columbus, USA).

### Sowing orders

At both sites, we established 20 square plots of 70 m^2^, using a randomized block design with four treatments replicated across five blocks per site. The experiment included two sequential species arrival treatments and two simultaneous species arrival treatments. In the sequential arrival treatments, one species group was sown 3 weeks before the other: (1) the fast‐group before the slow‐group (fast‐first) and (2) the slow‐group before the fast‐group (slow‐first). A 3‐week arrival interval was chosen to represent a realistic scenario in which species arrive or emerge within the same growing season, but at slightly different times due to phenological differences. Similar arrival intervals have been commonly used in grassland experiments (e.g. Delory, Weidlich, Kunz, et al., 2019; Dickson et al., 2012; Sarneel et al., 2016).

In the simultaneous arrival treatments, both species groups were introduced at the same time: (3) all species sown together at the early sowing moment (together‐early) and (4) all species sown together at the late sowing moment (together‐late). Thus, our experiment consisted of four arrival treatments: fast‐first, slow‐first, together‐early and together‐late. The early sowing moment was set at the beginning of each site's growing season, which was April 16, 2021 for the southern site (Lönnstorp) and June 9, 2021 for the northern site (Röbäcksdalen, see Appendix S1: Figure S3 for temperature sums at both sites).

Each plot was sown with a final density of 3000 seeds per m^2^, with 1500 seeds per m^2^ per group (i.e., slow‐germinating, fast‐germinating). The seed mixes were prepared by counting seeds by hand for low‐density species (30 seeds/m^2^ × 10 species = 300 seeds/m^2^) or weighting seeds for high‐density species (seed weight × 270 seeds/m^2^ × 10 species = total seeds in grams/m^2^, approximating 2700 seeds/m^2^).

Seeds were then mixed with approximately 200 mL sand (grout sand, Bygmaxx, Solna, Sweden) and broadcasted by hand under low‐wind conditions. To reduce impacts of weather that differed between the early and late sowing moments, we watered during dry conditions. In Lönnstorp, this required a single watering, whereas in Röbäcksdalen, plots were watered weekly for the first 3 weeks after the early sowing moment. Except for the first year, the plots were mowed yearly at the end of the growing season to simulate grazing, but they were not weeded.

### Field germination

To test our hypothesis, we treated germination speed as a functional trait, independent of the timing of species arrival during the season. In practice, however, this trait is likely influenced by environmental conditions. Therefore, we tested whether the species groups also exhibited relatively fast and slow germination under field conditions at both sowing moments. To do this, we repeated the germination tests that we did in the growth chambers at the experimental site in Röbäcksdalen. We sowed either 50 or 100 seeds (depending on availability, Appendix S1, Table S4) into 100‐cm^2^ plots and tracked their germination for 5 weeks after sowing, with five replicates per species. The experiment was shorter than the growth chamber trials because there was little germination after this amount of time. To align with the sowing orders described above, the sowing test was done twice with different starting dates; once early in the season (9th of June) and again 3 weeks later (30th of June). Germination rate and speed were calculated using the same methods as for the growth chamber trial, that is, germination rate was defined as the total proportion of seed that germinated, and germination speed (T50) as the time required to reach 50% of total germination.

### Plant community measurements

To quantify plant productivity, we harvested the standing biomass from a 0.25 m^2^ area in the center of each plot and weighted the biomass after drying at 60°C until it reached a stable weight. Species abundances were assessed at peak standing biomass (end of July in Lönnstorp and end of August in Röbäcksdalen) using a modified version of the pinpoint method (Jonasson, 1988). In each plot, two parallel transects were established, along which we placed 25 evenly‐spaced pins. For each pin, we recorded all species that touched the shaft of the pin and those species rooting within a 4‐cm radius around the base of the pin. Species abundances were expressed as the sum of pinpoint hits per plot, with a maximum value of 50 (two transects × 25 pins per plot).

### Statistical analyses

Differences in soil moisture content, nutrient levels, and pH were tested using two‐sided Student's *t*‐tests.

To test whether the fast‐ and slow‐germinating groups differed significantly in average germination speeds (T50), we used linear mixed‐effects models with speed group (fast or slow) and temperature (10°C and 15°C) as fixed effects, and species as a random effect (package *lme4*, Bates et al., 2015; R Core Team, 2023). We tested for differences in average germination rate in the growth chamber using a similar model, with the maximum germinated proportion as the response variable. To test whether the fast‐ and slow‐germinating groups also differed significantly in germination speed (T50) and rate in the field, we used two linear mixed‐effects models, one for T50 and the other for germination rate. Fixed effects included group (fast or slow) and sowing moment (early or late), while species names were included as a random effect. We further generated correlation plots (package *ggcorrplot*, Kassambara, 2023) to test the relationship between field and growth chamber germination speeds and rates in the field and in the growth chamber.

To test whether the sites (Lönnstorp and Röbäcksdalen) differed in plant productivity, we used a linear mixed‐effects model with biomass as the response variable, site and year as fixed effects, and plot nested within site as a random effect to account for repeated measurements over time.

To visualize vegetation changes over time at each site, we used nonmetric multidimensional scaling (NMDS; package *vegan*, Oksanen et al., 2022). The vegetation matrix consisted of species abundances (sum of pinpoint hits) of 20 plots over 4 years (80 rows). We ran the analysis both with non‐target species (i.e., species we did not sow) and without non‐target species in the data. Species that were absent in a given plot were given an abundance value of 0. We used pairwise Adonis tests to test whether plant community composition differed significantly between treatments within sites across years (package *adonis*, Martinez Arbizu, 2017).

To quantify the effects of sequential arrival, we calculated effect sizes (ES) for both early—(ES_early_, Equation 1) and late‐arriving species (ES_late_, Equation 2) within each block. The effect of arriving early (ES_early_) was calculated as the difference in species abundance between the sequential early‐arrival treatment (SEQ_early_) and the simultaneous early‐arrival treatment (SIM_early_), scaled by their sum (Equation 1). Likewise, the effect of arriving late (ES_late_) was calculated as the difference in species abundance between the sequential late‐arrival treatment (SEQ_late_) and the simultaneous late‐arrival treatment (SIM_late_), also scaled by their sum (Equation 2).

(1) $${\mathrm{ES}}_{\text{early}}=\frac{{\mathrm{SEQ}}_{\text{early}}‐{\mathrm{SIM}}_{\text{early}}}{{\mathrm{SEQ}}_{\text{early}}+{\mathrm{SIM}}_{\text{early}}}$$

(2) $${\mathrm{ES}}_{\text{late}}=\frac{{\mathrm{SEQ}}_{\text{late}}‐{\mathrm{SIM}}_{\text{late}}}{{\mathrm{SEQ}}_{\text{late}}+{\mathrm{SIM}}_{\text{late}}}$$

A positive effect size indicates that sequential arrival benefits the species, whereas a negative value indicates that sequential arrival is disadvantageous. For example, an ES_late_ of −0.5 indicates that a species' abundance is 50% lower when it arrives late compared to simultaneous arrival. Values near zero indicate little or no difference between sequential and simultaneous arrival scenarios. It should be noted that if a species is absent in both the sequential and simultaneous arrival treatment within a block, no effect size can be calculated for that species in that block. However, if a species is present in only one of the treatments (i.e., either in the sequential or in the simultaneous treatment), the priority effect is either −1 (indicating complete exclusion due to sequential arrival) or +1 (indicating complete facilitation due to sequential arrival).

To test whether priority effects are stronger when the fast‐germinating group arrives first (hypothesis 1) and whether species with lower initial density are more affected by priority effects (hypothesis 2), we built two linear mixed‐effects models: one with ES_late_ and another with ES_early_ as the response variable. For both models, fixed effects included arrival order, sowing density, site, and year, while random effects included species names and block nested within site.

Finally, to test whether the fast‐germinating group is generally more competitive than the slow‐germinating group (hypothesis 3), we built a linear mixed‐effects model with species abundances as the response variable. Fixed effects included site, group (fast or slow), arrival order, and year, while random effects included block nested within site.

For all linear mixed‐effects models, relevant pairwise comparisons were tested for significance using Tukey's HSD post hoc analysis.

## RESULTS

### Abiotic differences

Over the past decade, average air temperatures were higher in Lönnstorp (9.05 ± 7.17°C) compared to Röbäcksdalen (4.31 ± 9.76°C). The precipitation data showed that in the first year of the experiment (2021), the month of June was extremely dry in Lönnstorp (Appendix S1: Figure S3). Further, Röbäcksdalen experiences a thick snow cover during the winter months, typically from November to April.

Röbäcksdalen had a particle composition of 75% silt, 17% sand, 4.1% humus, and less than 4% clay, while Lönnstorp had a particle composition of 29% silt, 55% sand, 2.8% humus, and 13% clay. They are both classified as glacial till soils. The average moisture content of the soil was higher in Röbäcksdalen compared to Lönnstorp (0.23 ± 0.01% and 0.15 ± 0.01%, respectively, *t* = −53.49, df = 53.47, *p* < 0.001). The nutrient levels of the sites differed; Röbäcksdalen was higher in nitrogen, while Lönnstorp was higher in phosphorus (Appendix S1: Table S3). The pH was significantly higher in Lönnstorp compared to Röbäcksdalen (6.54 ± 0.11 and 5.89 ± 0.12, respectively, *t* = 17.51, df = 36.86, *p* < 0.001).

### Growth chamber germination

**FIGURE 3:**
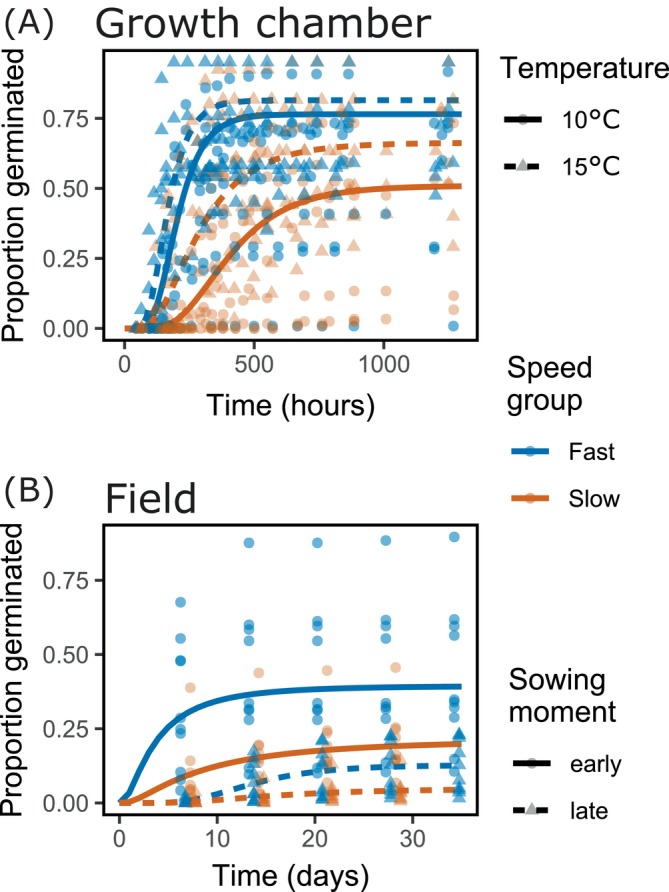
Germination data for both growth chamber (A) and field (B) germination tests. Symbols represent proportions of seed that germinated of one species at one timepoint, colored according to which group they are assigned to. Germination curves were fitted by time‐to‐event models. See Appendix S1: Sections S1.2 and S4.1 for germination curves of individual species.

On average, across 10°C and 15°C, the fast‐germinating group had a significantly lower mean T50 (216 ± 41 h) compared to the slow‐germinating group (592 ± 411 h, *F*_1,18.537_ = 9.74, *p* = 0.006) in the growth chamber (Figure 3A). Further, across the groups, germination speed was significantly slower at 10°C compared to 15°C (*F*_1,18.586_ = 14.42, *p* = 0.001). Germination rates were lower at 10°C compared to 15°C (*F*_1,17.223_ = 9.81, *p* = 0.006), but did not significantly differ between the fast‐ and slow‐germinating groups (*F*_1,17.704_ = 1.378, *p* = 0.256).

### Field germination

In the field, the slow‐germinating group had a significantly higher T50 than the fast‐germinating group (18.3 ± 14.6 and 10.7 ± 6.89 days; *F*_1,17,55_ = 5.10, *p* = 0.037, Figure 3B and Appendix S1: Table S5). In other words, the slow‐group germinated on average 8 days later than the fast‐group across both the early and late sowing moments. Germination speed was also affected by sowing moment; at the late sowing moment, the T50 of both groups was significantly higher compared to the early sowing moment (20.7 ± 13 and 7.81 ± 5.15, *F*_1,17,41_ = 19.92, *p* < 0.001).

Besides slower germination, the slow‐group also had a significantly lower germination rate compared to the fast‐group (0.12 ± 0.12 and 0.27 ± 0.24, *F*_1,18_ = 8.44, *p* = 0.010, Appendix S1: Figure S6). Germination rate was also affected by sowing moment; germination rate was significantly lower at the late sowing moment compared to the early sowing moment across species groups (0.1 ± 0.08 and 0.31 ± 0.22, *F*_1,18_ = 38, *p* < 0.001).

Correlation plots showed that species' T50 values in the growth chamber were predictive of species' T50 values in the field, but only at the early sowing moment. Germination rates in the growth chamber and the field were not correlated (Appendix S1: Figure S8). Overall, the fast‐group germinated both faster and more successfully compared to the slow‐group, and both groups showed slower and lower germination at the late sowing moment (Figure 3).

### Productivity

Overall, biomass production was lower in Lönnstorp compared to Röbäcksdalen. Across all years, the average biomass production was 420 ± 128 g/m^2^ and 780 ± 224 g/m^2^, respectively. Productivity increased significantly over the first 3 years in Röbäcksdalen, but remained stable in Lönnstorp (Appendix S1: Section S5).

### Vegetation development

The NMDS showed that sowing orders had distinct effects on plant community composition at the two sites. NMDS conducted with and without non‐target species showed similar results; however, treatments were visually more pronounced when non‐target species were included. Therefore, we present those results here, but see Appendix S1, Section S6.1 for results excluding non‐target species.

In Röbäcksdalen (NMDS: two dimensions, stress = 0.120), community composition shifted markedly over time and among sowing treatments. Across all treatments, a common pattern emerged: an initial set of non‐target species dominated early in the experiment, but declined over time, being replaced by other non‐target species. At the same time, some target species declined or disappeared, while a few became highly dominant. Despite these general trends, the sowing orders remained clearly distinct, indicating persistent differences in community composition throughout the experiment. By the final year, the two sequential sowing orders (fast‐first and slow‐first) were the most divergent, while the controls (together‐early and together‐late) fell in between. Pairwise Adonis confirmed that the slow‐first treatment led to a significantly different community composition compared to all other sowing orders (Appendix S1: Section S6.2). This differentiation was primarily driven by higher abundances of *Phalaris arundinacea*, *Poa pratensis*, and *Filipendula vulgaris* in the slow‐first treatment (Appendix S1: Figure S11).

**FIGURE 4:**
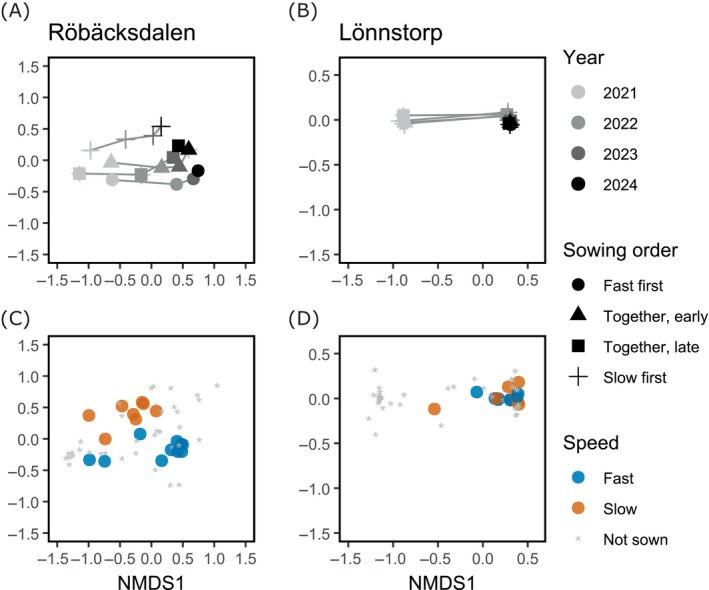
Changes in vegetation composition in Röbäcksdalen (A, B) and Lönnstorp (C, D) over time across sowing order treatments, visualized using non‐metric multidimensional scaling (NMDS) based on species abundances. In (A) and (B), symbols represent plot scores (averaged over sowing order and year) with standard errors. Lines connect treatment averages across successive years. In (B) and (D), symbols represent NMDS species scores based on pinpoint abundances (50 pins per plot) across years and treatments, that is, one symbol per species. Group indicates whether species belong to the fast‐ or slow‐germinating group and whether species were sown at a high or low initial density. Non‐target species, that is, species that were not sown, are indicated with asterisks. For analyses without non‐target species, see Appendix S1: Section S6.1.

In Lönnstorp (NMDS: two dimensions, stress = 0.059), community composition shifted markedly between the first and subsequent years. However, differences due to sowing order were minimal, as confirmed by pairwise Adonis analysis (Figure 4, Appendix S1: Section S6.2). The large initial shift was driven by a transient boom of “non‐target” species (i.e., species we did not sow). In the first year, these non‐target species dominated the plots, but in subsequent years, all plots became dominated by species from the fast‐germinating group, stabilizing for the remainder of the experiment.

**FIGURE 5:**
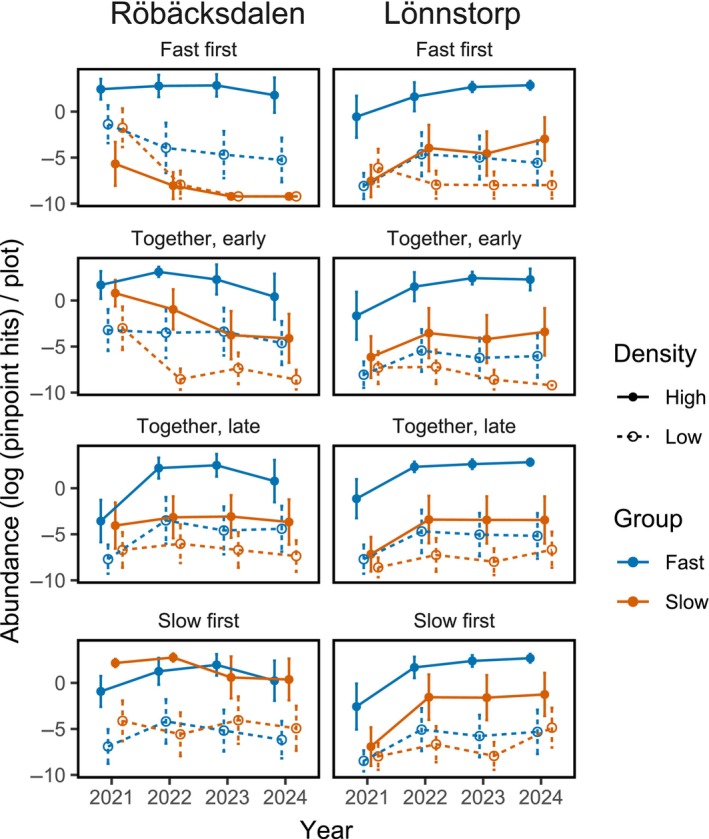
Average species abundances (*n* = 5 plots per dot) with standard errors grouped by high or low sowing density and fast or slow germination speed. Species abundances are measured as pinpoint hits per plots, with a maximum of 50 hits. Abundances are shown as log‐transformed values to improve visibility of very low abundances (see Appendix S1: Section S7.2).

The fast‐geminating group became dominant in all treatments, with the exception of the slow‐first treatment in Röbäcksdalen (Figure 5, see Appendix S1: Section S7.2 for full model output). Two of the 20 species sowed, *Pulsatilla vulgaris* and *Hypochaeris maculata*, were not recorded at any time at either site.

### Priority effects

**FIGURE 6:**
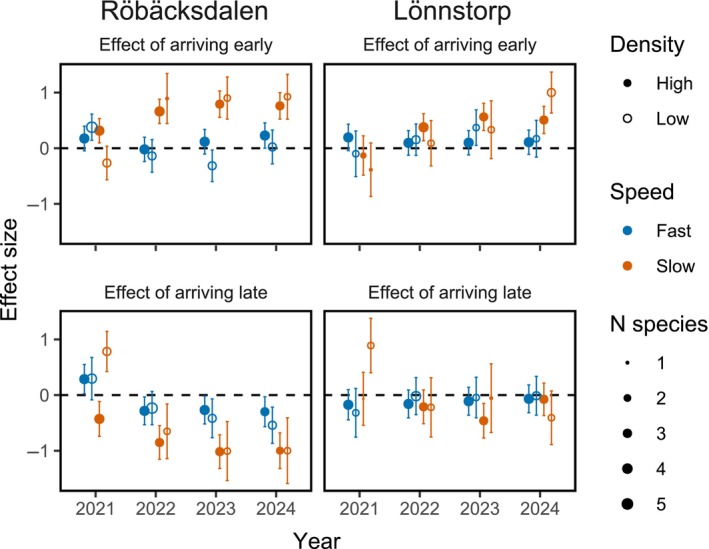
Estimated marginal means of effect sizes with confidence intervals calculated using *emmeans* based on two linear mixed‐effects models; one assessing the effect of arriving early (ES_early_) and the other assessing the effect of arriving late (ES_late_). The effect sizes used as the response variables in the models are calculated as the species abundance when arriving sequentially minus the species abundance when they arrive simultaneously, after which they are scaled. The size of the dots corresponds to the number of species associated with each mean. Values near zero indicate no effect of sequential arrival compared to simultaneous arrival, while positive values indicate a benefit and negative values indicate a disadvantage from either early or late arrival. See Appendix S1: Section S8 for full model outputs and pairwise comparison with Tukey's HSD.

Overall, the effect sizes of arriving early (ES_early_) were positive and increased over time, especially for the slow‐germinating group at both sites. In contrast, the effect sizes of arriving late (ES_late_) remained around zero throughout the experiment in Lönnstorp, but became increasingly negative over time in Röbäcksdalen for both species groups (Figure 6). The full model showed several significant interactions for both ES_early_ (Appendix S1: Table S13) and ES_late_ (Appendix S1: Table S17). Tukey's HSD post hoc tests showed that the benefit of early arrival was significantly greater for the slow‐germinating species in Röbäcksdalen compared to all other combinations of site and sowing order (Appendix S1: Table S14). Further, the benefit of early arrival for the slow‐germinating species strengthened over time in both sites. Fast‐germinating species in Röbäcksdalen also initially benefitted from early arrival, but this effect disappeared after the first year (Appendix S1: Table S15). The influence of seeding density on the benefit of early arrival was generally limited. However, in Lönnstorp, by the final year, low‐density slow‐germinating species had a higher ES_early_ than high‐density slow‐germinating species. Conversely, in Röbäcksdalen, during the first year, low‐density slow‐germinating species had a lower ES_early_ than the high‐density slow‐germinating species, though this effect did not persist (Appendix S1: Table S16).

For the effect of arriving late (ES_late_), Tukey's HSD post hoc tests showed that the slow‐germinating species in Röbäcksdalen experienced a stronger negative effect compared to all other combinations of site and sowing order (Appendix S1: Table S18). Moreover, these negative priority effects became stronger over time in Röbäcksdalen (Appendix S1: Table S19). Lastly, the low‐density slow‐germinating species experienced a positive effect of late arrival in both sites, but only during the first year (Appendix S1: Table S16).

## DISCUSSION

Priority effects can affect plant community dynamics via multiple pathways, such as niche preemption, niche modification, plant‐soil feedbacks, and soil legacies (Fukami, 2015; Grman & Suding, 2010). Our findings show that differences in germination traits, specifically the speed of germination after sowing, can modulate the strength of priority effects. In particular, we found that species that germinate slowly were more sensitive to priority effects. We found minimal effects of initial densities on the effect sizes of early and late arrival, indicating that species sown with low initial densities were not disproportionately affected by priority effects. We also found that the group of fast‐germinating species became dominant in the simultaneous arrival orders, indicating an overall benefit advantage of quick germination after sowing. Further, priority effects were site‐dependent, with priority effects being stronger in the more productive site with shorter growing seasons and longer, colder winters. In all, we found four out of the nine possible outcomes we hypothesized, ranging from no effects on early‐ and late‐arriving species to effects on both species groups. The treatments corresponded to Figure 2 as follows: fast‐first in Röbäcksdalen: 2C, slow‐first in Röbäcksdalen; 2B, fast‐first in Lönnstorp: 2E, and slow‐first in Lönnstorp: 2F. In other words, each of the four sequential arrival treatments (i.e., fast‐first and slow‐first in Röbäcksdalen and fast‐first and slow‐first in Lönnstorp) had unique outcomes, highlighting the influence of both species traits and site conditions on priority effects.

An arrival interval between species can be caused by differences in the arrival time of *seeds*, which can lead to priority effects. However, beyond the arrival time of seeds, differences in the timing of emergence may modulate priority effect strength. While the timing of emergence depends on environmental cues, it is also species‐dependent, causing species to emerge at different times (Blackford et al., 2020). Early emergence can enhance species' fitness (Verdú & Traveset, 2005) and may allow for more niche preemption or modification, leading to priority effects on later emerging species (Fukami, 2015). We defined the timing of emergence as the speed of germination after sowing, and hypothesized that sequential sowing of species groups with different mean germination speeds could lead to weaker or stronger priority effects depending on which group arrives first. In support of this hypothesis, we found that the slow‐germinating group experienced clear benefits of arriving early and was strongly negatively affected by arriving late. The fast‐germinating group only experienced negative effects of arriving late. We speculate that germinating slowly creates a competitive disadvantage; therefore, our results align with modern coexistence theory, which suggests that an opportunity for resource preemption is particularly important for competitively weaker species (Grainger et al., 2019). This is further supported by experimental evidence (Durbecq et al., 2023;Vaughn & Young, 2015; Werner et al., 2016), which all suggest that arriving early can be vital for the establishment of subordinate species. Our finding that stronger competitors were less affected by late arrival aligns with our previous studies (Sarneel et al., 2016; van Steijn et al., 2025). However, various studies, particularly ones including invasive species, contradict this by showing strong negative priority effects on otherwise highly competitive species (Delory, Weidlich, Kunz, et al., 2019; Durbecq et al., 2023; Lang et al., 2017).

Beyond the direct benefits of fast germination, we cannot rule out the possibility that fast‐germinating species are also more competitive in other aspects. In the field, the fast‐germinating group not only germinated more quickly but also achieved higher overall germination rates. This suggests that a number of early‐life traits, including germination speed and germination success, jointly contribute to the superior competitiveness of the fast‐germinating group compared to the slow‐germinating group. The importance of early‐life traits is further supported by the observation that when the slow‐germinating group gained a temporal advantage (i.e., was sown first), the competitive asymmetry between groups was eliminated in Röbäcksdalen and substantially reduced in Lönnstorp.

Moreover, we found that the benefits of early arrival strengthened over time, indicating a positive feedback in which early arrival enhances species performance in subsequent years. In Röbäcksdalen, the disadvantage of late arrival also became stronger over time, potentially due to the cold climate. Long winters may limit resource accumulation and overwintering success (Gioria et al., 2018), thereby amplifying the initial disadvantages in later years. Previous research supports the observation that the strength (Vaughn & Young, 2015; Werner et al., 2016) and even direction (Delory, Weidlich, von Gillhaussen, & Temperton, 2019) of priority effects can change over time.

Our second hypothesis states that species with a low initial seed density benefit more from arriving early and experience a larger disadvantage from arriving late. Our findings did not support this hypothesis, as the few effects of initial density we found were weak and transient. Our results therefore fall in line with von Gillhaussen et al. (2014), who found that priority effects were not dependent on initial seed density when tested in the greenhouse. Our study builds upon their work by demonstrating that even in the field and with asymmetrical initial densities between species, priority effects were still neither stronger nor weaker based on density differences. However, the effects of sowing density may have been confounded by variations in field germination rates. For example, the 80% germination rate observed in the low‐density seeded *Dianthus deltoides* would result in only slightly lower seedling numbers compared to the 10% germination rate in the high‐density sown *Campanula persicifolia*. Future research on how initial density influences priority effect strength may benefit from using early biomass as a proxy for realized initial density rather than sown seed density (Zou & Rudolf, 2023).

We found that the simultaneously sown communities became dominated by species from the fast‐germinating group. This falls in line with our third hypothesis that a small “built in” arrival interval due to differences in the timing of emergence can cause within‐year priority effects, also referred to as *seasonal priority effects* (Wolkovich & Cleland, 2014). This finding further highlights the importance of phenology, which plays a crucial role in shaping contrasting ecological strategies, such as early‐emerging, fast‐growing species versus late‐emerging, slow‐growing species (Sun & Frelich, 2011). In turn, species phenologies affect community structure and species' ability to coexist, which can be disrupted by climate change (Rudolf, 2019). Research on invasive species further underscores the significance of interactions between phenologies, as many invasive species emerge earlier in the season, often gaining a competitive advantage over native species through priority effects (Dickson et al., 2012; Stevens & Fehmi, 2011; Vaughn & Young, 2015).

Further, the outcomes of the arrival treatments were site‐dependent; both sequential arrival orders (i.e., fast‐germinating species first and slow‐germinating species first) played out differently in each site. The sites differed in various ways: Röbäcksdalen had silty soil with higher nitrogen content and higher moisture levels, which together likely contributed to the higher productivity of this site. Lönnstorp had sandy soils that were drier and with a lower nitrogen content. We observed stronger priority effects in Röbäcksdalen, which supports previous research suggesting that priority effects are stronger in environments with higher productivity or nutrient availability (Chase, 2003; Kardol et al., 2013, resp.). Several field experiments further support this. Fry et al. (2017) found stronger priority effects in grassland mesocosms with nutrient‐rich clay soils compared to nutrient‐poorer sandy soils, and Young et al. (2015) similarly found weaker priority effects for invasive species abundance in their least productive prairie site compared to their most productive site. Conversely, the order of species arrival did not strongly influence community trajectories in Lönnstorp, since the fast‐germinating group quickly established dominance and maintained it throughout the experiment there. This supports the idea that as the importance of the competitive hierarchy increases, arrival order starts to matter less (Fukami, 2015).

To our knowledge, this is the first study to test priority effects across highly contrasting sites using the same set of species. Although our two study sites differed in multiple ways, limiting our ability to isolate specific site effects, the pronounced differences in priority effects between them offer a strong foundation for future research. Further studies designed to disentangle the individual contributions of climatic, soil, and biotic factors will be needed for a more precise understanding of how site conditions shape priority effects.

## CONFLICT OF INTEREST STATEMENT

The authors report no conflict of interest regarding this work.

## Supporting information


Appendix S1.

## Data Availability

Data and code (van Steijn, 2025) are available in Zenodo at https://doi.org/10.5281/zenodo.15363824.
