# Supplementary material for: Germination speed modulates priority effects: Evidence from a large‐scale field study
Source: Ecology. 2026 Jan 23;107(1):e70291. doi: 10.1002/ecy.70291 (PMC12830169; doi:10.1002/ecy.70291)
Supplement: Supplementary file 1 — Appendix S1. [file ECY-107-e70291-s001.pdf]

Germination speed modulates priority effects: Evidence from a large-scale field study

Tamara L.H. van Steijn[2], Paul Kardol[2,3], Roland Jansson[1], and Judith M. Sarneel[1]

[1]Umeå University, Department of Ecology and Environmental Science, Umeå, Sweden
[2]Swedish University of Agricultural Sciences, Department of Forest Ecology and Management, Umeå, Sweden
[3]Swedish University of Agricultural Sciences, Department of Forest Mycology and Plant, Uppsala, Sweden

Appendix S1

Corresponding author:
Tamara van Steijn
Tamara.van.steijn@slu.se

Open research statement:
Data and code is openly available on Zenodo via the following link:
https://zenodo.org/records/15363824

Keywords: boreal, plant competition, plant community assembly, phenology, priority effects, seed density, germination speed

Contents

S1 Species selection **3**
S1.1 Species table . . . 3
S1.2 Growth chamber germination curves . . . 4

S2 Density treatment **6**

S3 Site descriptions **6**
S3.1 Soil analysis . . . 6
S3.2 Weather 2021 . . . 7

S4 Field germination **9**
S4.1 Field germination curves . . . 9
S4.2 Germination speed . . . 11
S4.3 Germination rate . . . 12
S4.4 Indoor and outdoor germination correlations . . . 13

S5 Biomass per site **13**

S6 Adonis **15**
S6.1 Without non-target species 15
S6.2 With non-target species 16

S7 Abundances **17**
S7.1 Final year abundances 17
S7.2 Abundance model 19

S8 Priority effects **21**
S8.1 The effect of arriving early 21
S8.2 The effect of arriving late 23

S1 Species selection

S1.1 Species table

Table S1: The first column shows the initial species selection, of which some are indicator species of high value meadow and pasture land (Jordbruksverket 2016) and others are common across Sweden (SLU Artdatabanken). We discarded species if had a germination rate less than 20% and based our final selection on seed availability. We calculated the time needed to reach 50% of total germinated (T50) and grouped species in fast- and slow-germinating groups (Fig. S2).

Species	Indicator	Germination >20%	Final Selection	T50 at 10°C
Agrostis capillaris	-	Yes	Yes	260
Antennaria dioica	Yes	Yes	Yes	276
Briza media	Yes	Yes	Yes	399
Calamagrostis epigejos	-	Yes	Yes	396
Campanula persicifolia	Yes	Yes	Yes	418
Cardamine pratensis	Yes	Yes	Yes	265
Deschampsia cespitosa	-	Yes	Yes	245
Deschampsia flexuosa	-	-	-	-
Dianthus deltoides	Yes	Yes	Yes	173
Festuca rubra	-	Yes	Yes	253
Filipendula vulgaris	Yes	Yes	Yes	394
Galium verum	Yes	Yes	Yes	154
Hypochaeris maculata	Yes	Yes	Yes	303
Lathyrus pratensis	-	Yes	-	-
Lathyrus sylvestris	-	Yes	-	-
Leucanthemum vulgare	Yes	Yes	Yes	211
Lotus corniculatus	-	Yes	-	-
Lychnis flos-cuculi	Yes	Yes	-	-
Phalaris arundinacea	-	Yes	Yes	508
Pimpinella saxifraga	Yes	-	-	-
Plantago media	Yes	Yes	Yes	98*
Poa alpina	Yes	Yes	Yes	174
Poa pratensis	-	Yes	Yes	554
Poa trivialis	-	Yes	-	-
Potentilla erecta	Yes	-	-	-
Primula farinosa	Yes	-	-	-
Primula veris	Yes	-	-	-
Pulsatilla vulgaris	Yes	Yes	Yes	1132
Rhinanthus angustifolius	Yes	-	-	-
Sedum acre	Yes	Yes	Yes	212
Succisa pratensis	Yes	Yes	Yes	1537
Trifolium pratense	-	Yes	-	-
Trifolium repens	-	Yes	-	-
Trollius europaeus	-	-	-	-
Vicia cracca	-	Yes	-	-

*T50 for 15°C, since the model did not compute for 10°C.

S1.2 Growth chamber germination curves

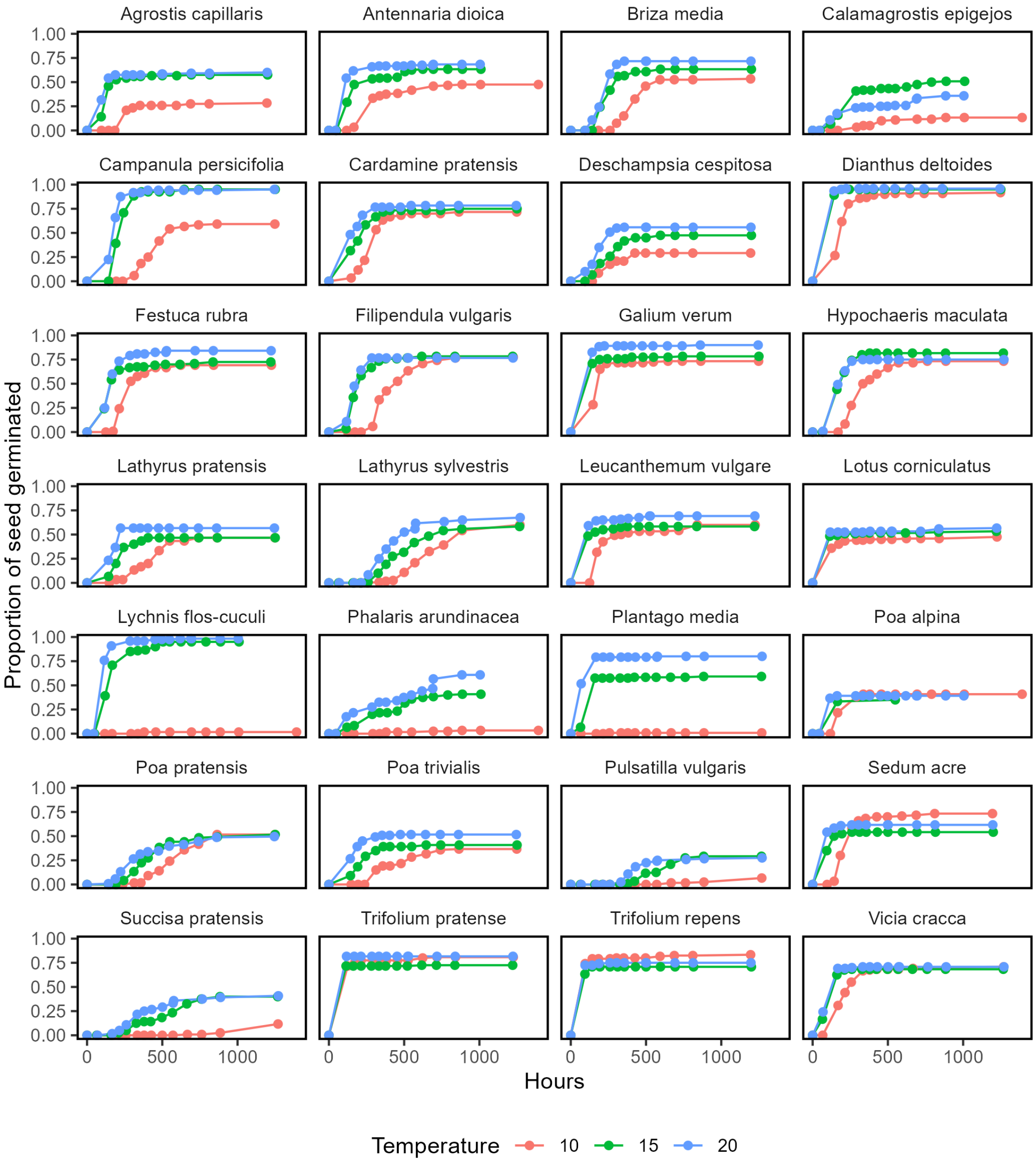

Figure S1: Germination data for each species in the growth chambers at different temperatures. Germinated proportions are based on pooled species data (i.e., summed over all replicates for each temperature).

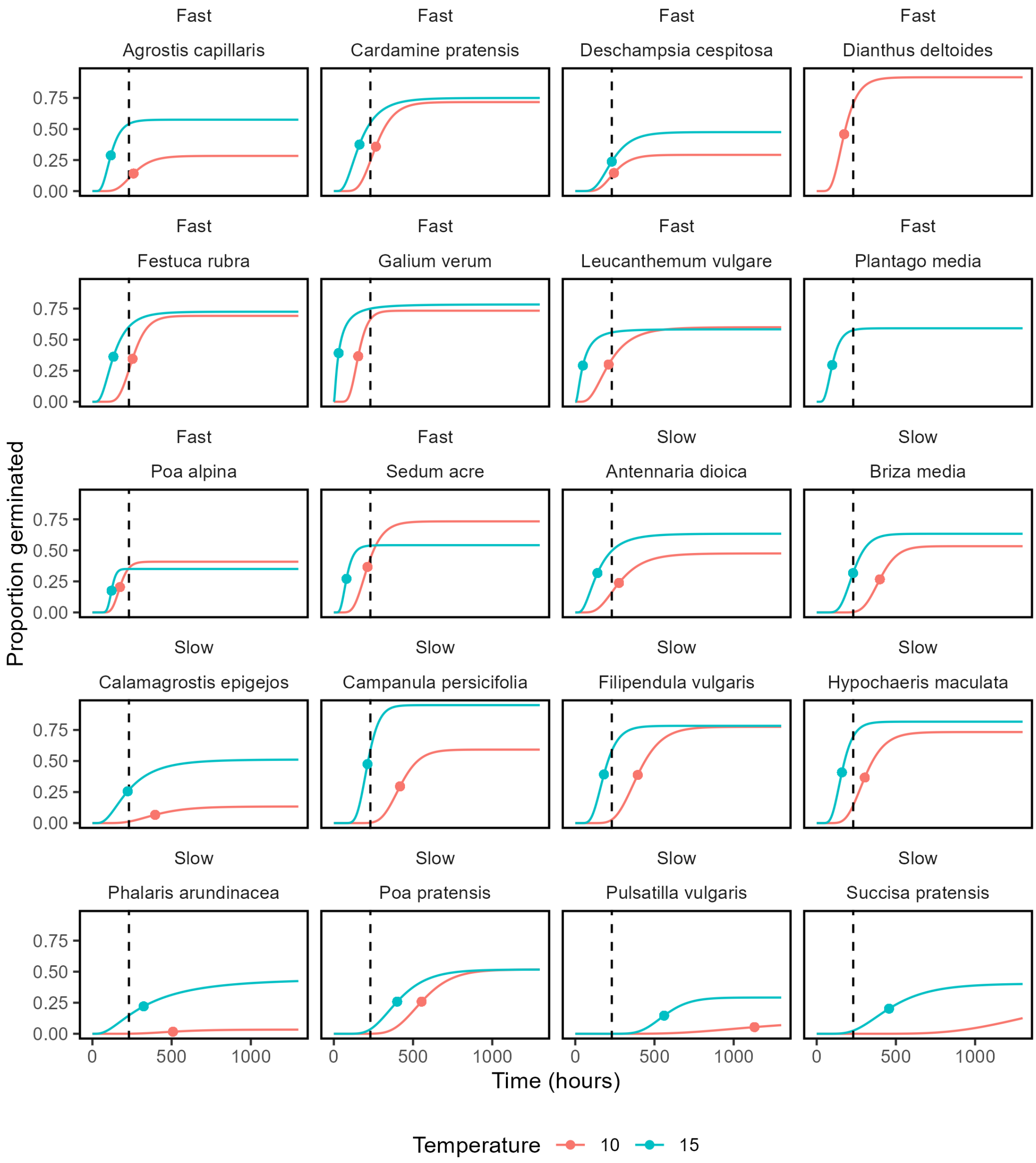

Figure S2: Germination curves based on predictions by time-to-event models using the data from Fig. S1. For the grouping of germination speed, we focussed on 10 and 15°C, as this would be more similar to field conditions than 20°C. The dashed line represents the median of all germination speeds depicted. Dots indicate at which hour T50 is reached. The final group selections are indicated by Fast and Slow. Germination for *Plantago media* at 10°C was too low, and germination for *Dianthus deltoides* at 15°C was too fast to compute models.

S2 Density treatment

Table S2: Plant species used in the experiment in alphabetical order with the sowing density and the reasoning we used to assign species to their sowing density.

Species	Sowing density	Reasoning
Agrostis capillaris (L.)	High	Invasive in some areas and strong vegetative spread
Antennaria dioica (L. Gaertn.)	High	Strong vegetative spread
Briza media (L.)	Low	Small and weak vegetative spread
Calamagrostis epigejos (L.)	Low	*Assigned to balance group*
Campanula persicifolia (L.)	High	Tall and strong vegetative spread
Cardamine pratensis (L.)	Low	Small and weak vegetative spread
Deschampsia cespitosa (L.)	Low	Weak vegetative spread
Dianthus deltoides (L.)	Low	Weak vegetative spread
Festuca rubra (L.)	High	Able to thrive in many environments
Filipendula vulgaris (L.)	High	Tall with strong vegetative spread
Galium verum (L.)	High	Able to thrive in many environments
Hypochaeris maculata (L.)	Low	Listed as near threatened
Leucanthemum vulgare (Lam.)	High	Invasive in certain areas
Phalaris arundinacea (L.)	High	Invasive in certain areas
Plantago media (L.)	High	*Assigned to balance group*
Poa alpina (L.)	Low	Naturally uncommon
Poa pratensis (L.)	High	Invasive in some areas and strong vegetative spread
Pulsatilla vulgaris (L.)	Low	Listed as vulnerable
Sedum acre (L.)	Low	Low and slow growing
Succisa pratensis (L.)	Low	Weak vegetative spread

S3 Site descriptions

S3.1 Soil analysis

Soil extracts were made by shaking 5 g of the soil mixture in 25 ml of water followed by filtration using 0.45 µm Filtropur.

Dissolved and total nutrients is analyzed by measuring color on a photometer in a segmented flow analyzer, after various reagents have been added. NO_3 (NO_3 and NO_2) after reagents and samples passed a copperized Cd reduction coil to form an azo dye [method: MT3B Q-126-12 Rev 1]. NH_4 with the salicylate method [method: Q-033-04 Rev. 8] and PO_4 with the molybdenum blue method [method: MT3A Q-125-12 Rev 1].

Total nutrients analyzed in the same manner after having passed an on-line digestion step (alkaline acidic persulfate method) at 110 ºC and 0.9 MPa [method: Q-115-10 Rev. 4]. Nutrients have been analyzed on a QuAAtro 39 from Seal Analytical. See table S3 for results.

Some nutrient values were extremely large, indicating some samples were faulty. We removed three outliers because they were 2.5 times the median.

Table S3: Nutrient levels for different sites with standard deviations and results of two-sided t-tests.

Nutrient	Lönnstorp	Röbäcksdalen	t-value	p-value
NO_3-N (mg/kg)	1962 ± 768	2944 ± 709	-4.1414	<**0.001**
NH_4-N (mg/kg)	81 ± 34	112 ± 43	-3.2082	<**0.001**
PO_4-P (mg/kg)	151 ± 59	52 ± 23	7.0039	<**0.001**

S3.2 Weather 2021

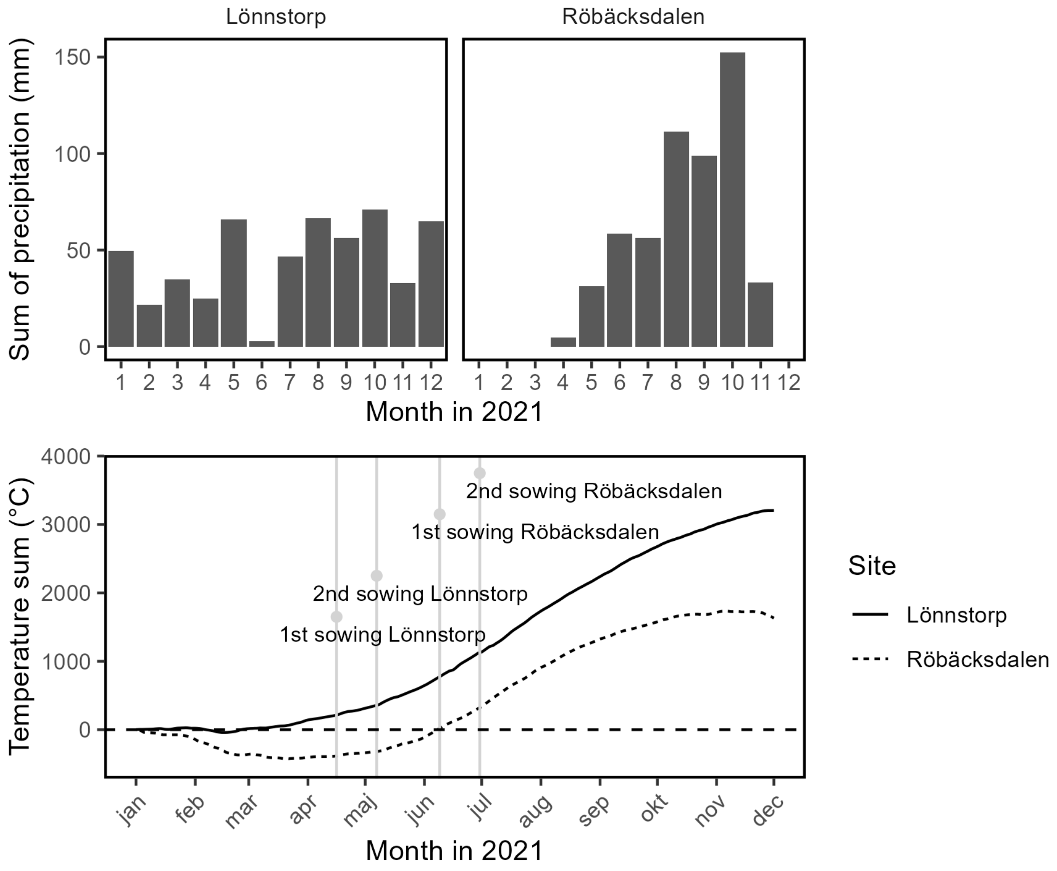

Figure S3: Precipitation and temperature sum data of the starting year (2021) for both sites. The growing season starts later in Röbäcksdalen and overall, the temperature sum is lower at this location.

Species	Seeds sown
Pulsatilla vulgaris	100
Phalaris arundinacea	100
Sedum acre	100
Plantago media	100
Cardamine pratensis	50
Festuca rubra	50
Filipendula vulgaris	50
Calamagrostis epigejos	100
Hypochaeris maculata	50
Briza media	100
Antennaria dioica	100
Deschampsia cespitosa	100
Agrostis capillaris	100
Leucanthemum vulgare	100
Galium verum	50
Dianthus deltoides	50
Succisa pratensis	100
Poa alpina	100
Poa pratensis	100
Campanula persicifolia	50

Table S4: List of species and number sown in field germination trials.

S4 Field germination

S4.1 Field germination curves

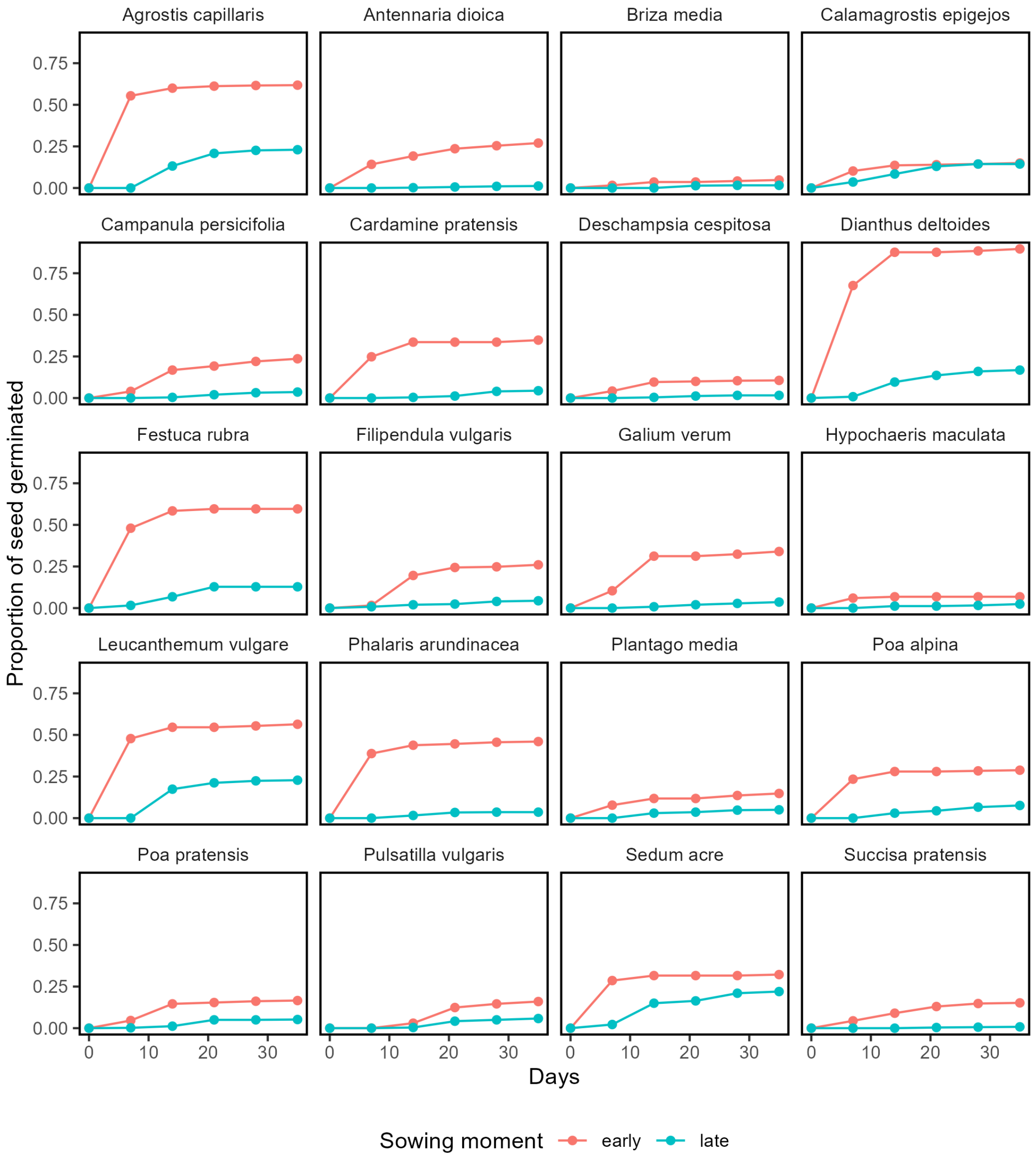

Figure S4: Germination data for each species in the field, sown either early in the season (9th of June 2021) or late (30th of June 2021). Germinated proportions are based on pooled species data (i.e., summed over all replicates for each sowing moment).

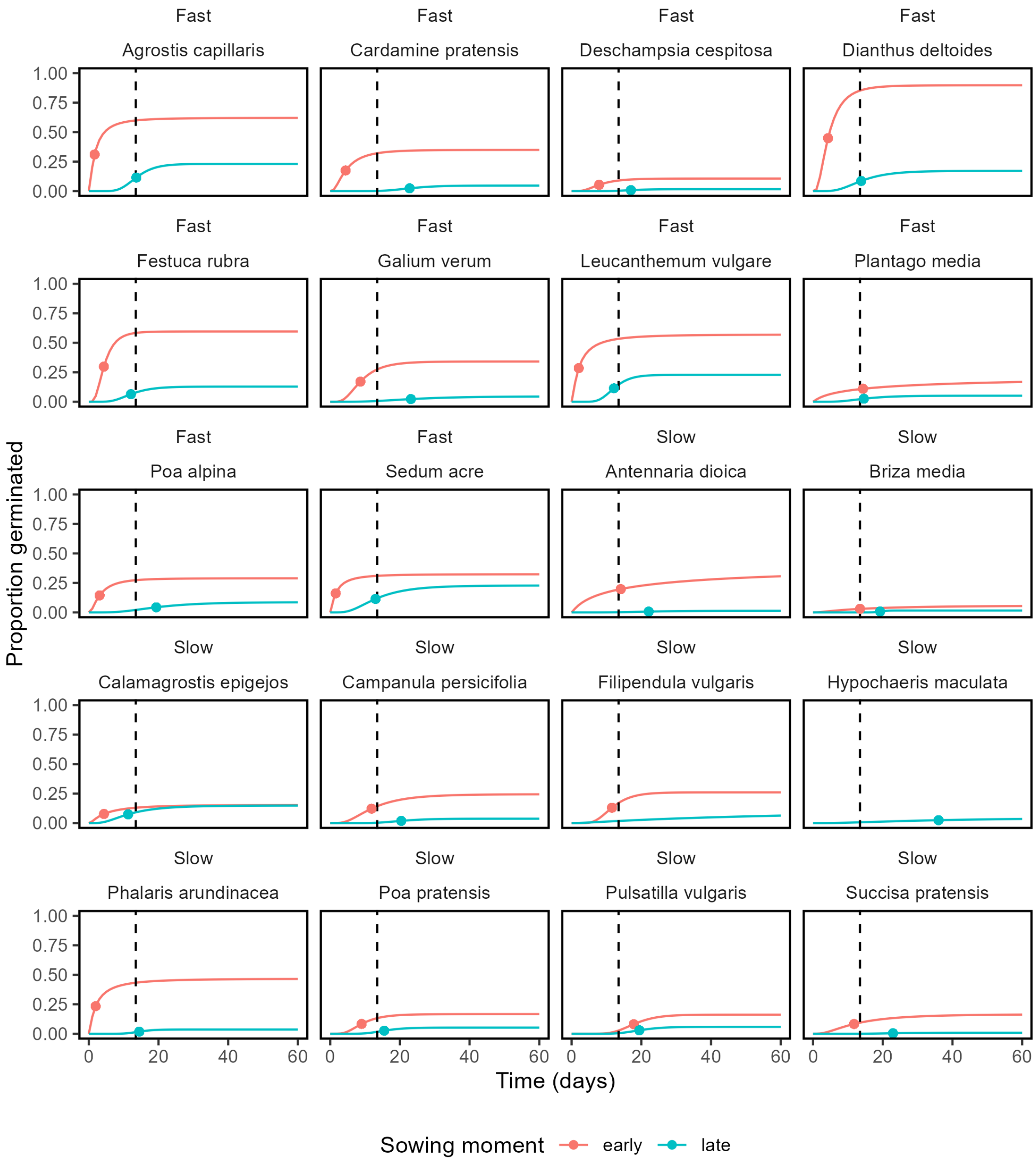

Figure S5: Germination curves based on prediction by time-to-event models using the data from Fig. S4, with the two different sowing moments indicated by line color. The dashed line represents the median of all outdoor germination speeds. Dots indicate at which day T50 is reached.

S4.2 Germination speed

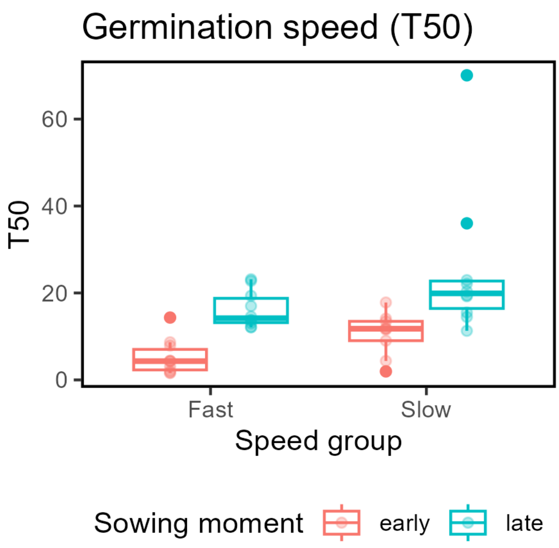

Figure S6: The germination speeds (T50) for the species in each group (fast or slow) and for each sowing moment (early or late) in the field at the Röbäcksdalen site. The n for each bar is 10 species, for which averages are calculated over 5 replicates.

Table S5: Type III Analysis of Variance Table for T50. The fast-germinating germinated faster that the slow-germinating group. Futher, both groups germinated slower at the late sowing moment.

**Effect**	**Sum Sq**	**Mean Sq**	**NumDF**	**DenDF**	**F value**	**Pr(>F)**
Speed	395.77	395.77	1	17.555	5.1026	**0.037**
Sowing moment	1545.32	1545.32	1	17.413	19.9234	<**0.001**
Speed x Sowing moment	28.05	28.05	1	17.413	0.3616	0.555

S4.3 Germination rate

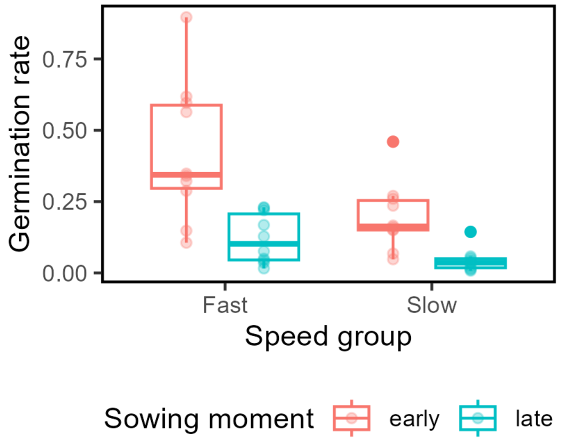

Figure S7: The average maximum proportion of seed that germinated (i.e., germination rate) for the species in each group, for both the early and late sowing moment in the field in Röbäcksdalen. The n for each bar is 10 species, for which averages are calculated over 5 replicates.

Table S6: Type III Analysis of Variance Table for the germination rate. The slow-group germinated less that the fast-germinating group, and both groups germinated less at the late sowing moment compared to the early sowing moment. P values below 0.05 are indicated in bold.

**Effect**	**Sum Sq**	**Mean Sq**	**NumDF**	**DenDF**	**F value**	**Pr(>F)**
Speed	0.11610	0.11610	1	18	8.4403	**0.010**
Sowing moment	0.52212	0.52212	1	18	37.9564	<**0.001**
Speed x sowing moment	0.05550	0.05550	1	18	4.0348	0.056

S4.4 Indoor and outdoor germination correlations

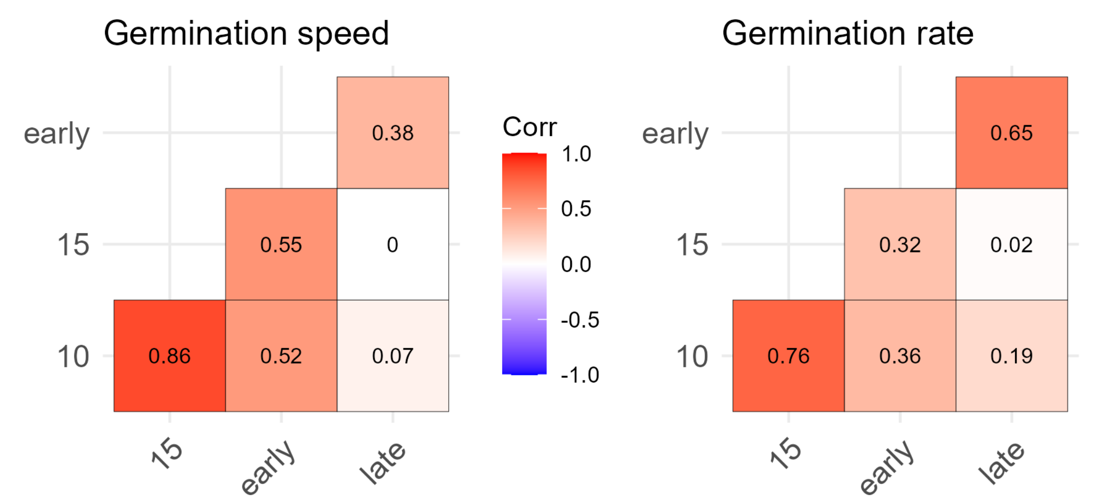

Figure S8: Pearson correlations coefficients between total germination rates and speeds in the growth chamber (at 10 and 15°C) and in the field the site Röbäcksdalen (sown either early or late). Germination speed in the growth chamber predicted germination speed when sown early in the field, but not when sown late in the field.Germination rate in the growth chamber did not strongly predict germination rate in the field. Higher color saturation indiate stronger correlations. n = 20 for each correlation (average values for each species).

S5 Biomass per site

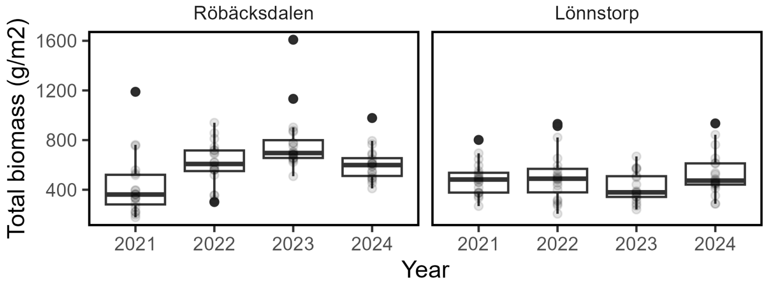

Figure S9: Biomass over time per site. Biomass is harvested in 0.25 m^2 subplots.

Table S7: Type III Analysis of Variance Table with Satterthwaite's Method for results of a linear mixed effects model, testing the effect of site, year and their interaction on total biomass. The significant interaction shows that biomass changes over time in Röbäcksdalen, but is stable in Lönnstorp.

**Variable**	**Df**	**Sum Sq**	**Mean Sq**	**F value**	**Pr(>F)**
Site	1	19401	19401.1	11.238	**0.002**
Year	3	27232	9077.4	5.258	**0.002**
Site x Year	3	53721	17907.1	10.372	<**0.001**
Residuals	149				

S6 Adonis

S6.1 Without non-target species

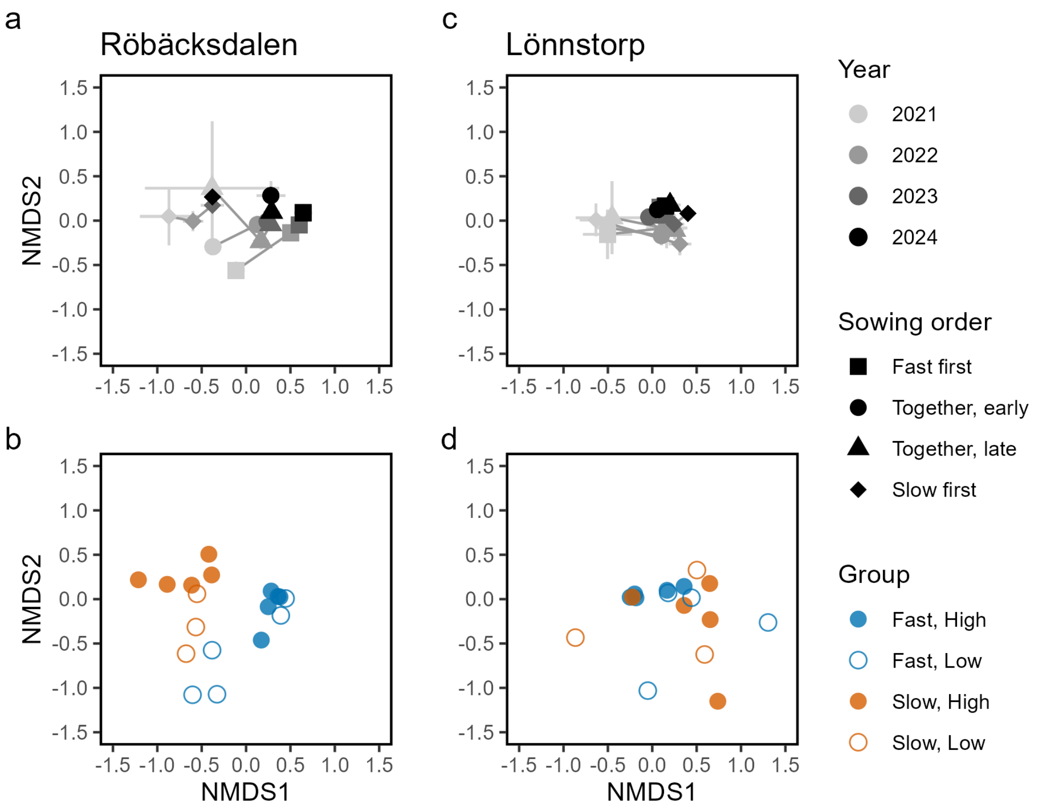

Figure S10: Changes in vegetation composition in Röbäcksdalen (a,b) and Lönnstorp (c,d) over time across sowing order treatments, visualized using NMDS (two dimensions, stress = 0.126). In a and c, symbols represent plot scores (averaged over sowing order and year) with standard errors. Non-target species, i.e. species we did not sow, were excluded. Lines connect treatment averages across successive years. In b and d, symbols represent NMDS species scores based on pinpoint abundances (50 pins per plot). Group indicates whether species belong to the fast- or slow-germinating group and whether species were sown at a high or low initial density.

Table S8: Pairwise Adonis results for Röbäcksdalen (RB) and Lönnstorp (LT, df = 1) without non-target species in the data. The results show that the community in the slow-first plots differ significantly from all other communities in Röbäcksdalen. In Lönnstorp there are no significant differences between the communities.

	Pairs	Sums of Sq	F.Model	R^2	p-value	p-adjusted
RB	Together, early vs Together, late	0.1074	3.9420	0.0940	0.031	0.186
	Together, early vs Slow-first	0.7453	48.4066	0.5602	0.001	**0.006**
	Together, early vs Fast-first	0.0911	6.3255	0.1427	0.007	0.042
	Together, late vs Slow-first	0.5351	19.9281	0.3440	0.001	**0.006**
	Together, late vs Fast-first	0.1117	4.3197	0.1021	0.023	0.138
	Slow-first vs Fast-first	1.0679	76.2997	0.6675	0.001	**0.006**
LT	Fast-first vs Together, early	0.0254	0.8410	0.0217	0.354	1.000
	Fast-first vs Slow-first	0.0955	2.5663	0.0633	0.100	0.600
	Fast-first vs Together, late	0.0274	0.6825	0.0176	0.427	1.000
	Together, early vs Slow-first	0.0781	2.0607	0.0514	0.165	0.990
	Together, early vs Together, late	0.0363	0.8909	0.0229	0.341	1.000
	Slow-first vs Together, late	0.0364	0.7611	0.0196	0.398	1.000

S6.2 With non-target species

Table S9: Pairwise Adonis results for Röbäcksdalen (RB) and Lönnstorp (LT, df = 1) with non-target species in the data. The results show that the community in the slow-first plots differ significantly from all other communities in Röbäcksdalen. In Lönnstorp there are no significant differences between the communities.

Site	Pairs	Sums of Sq	F.Model	R^2	p-value	p-adjusted
RB	Together, early vs Together, late	0.1035	4.2131	0.0998	0.042	0.252
	Together, early vs Slow-first	0.4118	24.2939	0.3900	0.001	**0.006**
	Together, early vs Fast-first	0.0551	3.0052	0.0733	0.072	0.432
	Together, late vs Slow-first	0.2154	9.6015	0.2017	0.001	**0.006**
	Together, late vs Fast-first	0.1087	4.5640	0.1072	0.024	0.144
	Slow-first vs Fast-first	0.5743	35.4260	0.4825	0.001	**0.006**
LT	Fast-first vs Together, early	0.0103	0.2892	0.0076	0.670	1.000
	Fast-first vs Slow-first	0.0444	1.1762	0.0300	0.290	1.000
	Fast-first vs Together, late	0.0115	0.2837	0.0074	0.688	1.000
	Together, early vs Slow-first	0.0297	0.7706	0.0199	0.377	1.000
	Together, early vs Together, late	0.0123	0.2977	0.0078	0.659	1.000
	Slow-first vs Together, late	0.0175	0.4056	0.0106	0.588	1.000

S7 Abundances

S7.1 Final year abundances

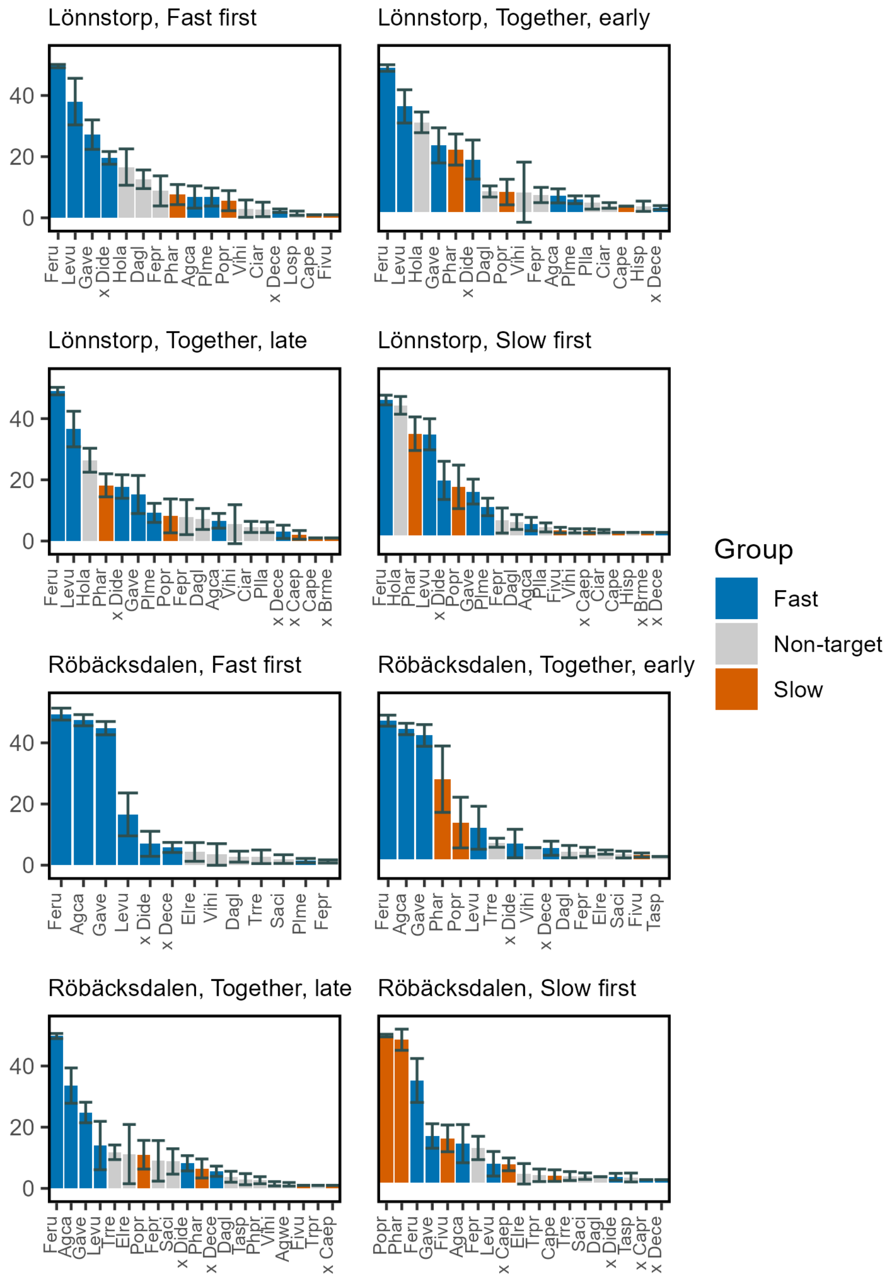

Figure S11: Final year average abundances based on pinpoint data (maximum abundance is 50). Species sown at a low density are marked with an X. See Table S10 for species abbreviations.

Table S10: Species names abbreviations.

**Species**	**Abbreviation**
Achillea millefolium	Acmi
Agrostis capillaris	Agca
Agrostis weed	Agwe
Brassica napus	Brna
Briza media	Brme
Calamagrostis epigejos	Caep
Campanula persicifolia	Cape
Cardamine pratensis	Capr
Cirsium arvense	Ciar
Dactylis glomerata	Dagl
Deschampsia cespitosa	Dece
Dianthus deltoides	Dide
Elymus repens	Elre
Festuca pratensis	Fepr
Festuca rubra	Feru
Filipendula vulgaris	Fivu
Galium verum	Gave
Geranium sylvaticum	Gesy
Hieracium sp.	Hisp
Holcus lanatus	Hola
Jacobaea vulgaris	Javu
Leucanthemum vulgare	Levu
Lotus sp.	Losp
Medicago sativa	Mesa
Phalaris arundinacea	Phar
Phleum pratense	Phpr
Plantago lanceolata	Plla
Plantago media	Plme
Poa pratensis	Popr
Rumex crispus	Rucr
Salix cinerea	Saci
Succisa pratensis	Supr
Tanacetum vulgare	Tavu
Taraxacum sp.	Tasp
Trifolium pratensis	Trpr
Trifolium repens	Trre
Tussilago sp.	Tusp
Vicia hirsuta	Vihi

S7.2 Abundance model

Table S11: Type III Analysis of Variance Table with Satterthwaite's Method. Based on a linear mixed effects models using species abundances as the response variable and group (fast- or slow-germinating species), sowing order (fast-first, slow-first, together-late, together-early), site, year and density as fixed effects and species as random effects and block/site as nested random effects.

**Variable**	**SumSq**	**MeanSq**	**NumDF**	**DenDF**	**F-value**	**P-Value**	**Tukey**
Group (G)	183.7	183.7	1	14	3.856	0.070	
Order (O)	1993.5	664.5	3	2738	13.952	<**0.001**	
Site (S)	3352.9	3352.9	1	2738	70.397	<**0.001**	
Density (D)	420.3	420.3	1	14	8.825	**0.010**	
Year (Y)	12770.5	4256.8	3	2738	89.376	<**0.001**	
Group x Order	17777.4	5925.8	3	2738	124.417	<**0.001**	
Group x Site	70.8	70.8	1	2738	1.486	0.223	
Order x Site	958.1	319.4	3	2738	6.706	<**0.001**	
Group x Density	96.0	96.0	1	14	2.016	0.178	
Order x Density	1344.3	448.1	3	2738	9.409	<**0.001**	
Site x Density	844.9	844.9	1	2738	17.739	<**0.001**	
Group x Year	7595.7	2531.9	3	2738	53.160	<**0.001**	
Order x Year	1368.4	152.0	9	2738	3.192	**0.001**	
Site x Year	710.3	236.8	3	2738	4.971	**0.002**	
Density x Year	8148.1	2716.0	3	2738	57.025	<**0.001**	
G x O x S	6811.6	2270.5	3	2738	47.672	<**0.001**	
G x O x D	10948.9	3649.6	3	2738	76.627	<**0.001**	
G x S x D	5.9	5.9	1	2738	0.123	0.726	
O x S x D	443.3	147.8	3	2738	3.103	**0.026**	
G x O x Y	1520.0	168.9	9	2738	3.546	<**0.001**	
G x S x Y	1228.9	409.6	3	2738	8.600	<**0.001**	
O x S x Y	315.0	35.0	9	2738	0.735	0.677	
G x D x Y	3857.9	1286.0	3	2738	27.000	<**0.001**	
O x D x Y	417.2	46.4	9	2738	0.973	0.460	
S x D x Y	116.0	38.7	3	2738	0.812	0.487	
G x O x S x D	3114.2	1038.1	3	2738	21.795	<**0.001**	S12
G x O x S x Y	839.3	93.3	9	2738	1.958	**0.040**	
G x O x D x Y	674.8	75.0	9	2738	1.574	0.117	
G x S x D x Y	282.5	94.2	3	2738	1.977	0.115	
O x S x D x Y	47.3	5.3	9	2738	0.110	0.999	
G x O x S x D x Y	176.4	19.6	9	2738	0.412	0.930	

Table S12: TukeyHSD posthoc analysis on the third order interaction between germination group, sowing treatment, site (Röbäcksdalen (RD) and Lönnstorp (LT)) and sowing density, within treatments, not across.

Order	Site	Contrast	Estimate	SE	df	t-ratio	p-value
Fast first	LT	Fast High - Slow High	18.04	5.2047	14.9	3.466	**0.016**
		Fast High - Fast Low	18.20	5.2047	14.9	3.497	**0.015**
		Fast High - Slow Low	20.13	6.0099	14.9	3.350	**0.021**
		Slow High - Fast Low	0.16	5.2047	14.9	0.031	1.000
		Slow High - Slow Low	2.09	6.0099	14.9	0.348	0.985
		Fast Low - Slow Low	1.93	6.0099	14.9	0.322	0.988
Together-late	LT	Fast High - Slow High	13.59	5.2047	14.9	2.611	0.083
		Fast High - Fast Low	17.36	5.2047	14.9	3.335	**0.021**
		Fast High - Slow Low	18.99	6.0099	14.9	3.160	**0.030**
		Slow High - Fast Low	3.77	5.2047	14.9	0.724	0.886
		Slow High - Slow Low	5.40	6.0099	14.9	0.899	0.806
		Fast Low - Slow Low	1.63	6.0099	14.9	0.272	0.993
Together-late	LT	Fast High - Slow High	13.27	5.2047	14.9	2.550	0.092
		Fast High - Fast Low	15.45	5.2047	14.9	2.968	**0.043**
		Fast High - Slow Low	17.15	6.0099	14.9	2.854	0.053
		Slow High - Fast Low	2.18	5.2047	14.9	0.419	0.974
		Slow High - Slow Low	3.88	6.0099	14.9	0.646	0.915
		Fast Low - Slow Low	1.70	6.0099	14.9	0.283	0.992
Slow first	LT	Fast High - Slow High	7.15	5.2047	14.9	1.374	0.534
		Fast High - Fast Low	13.65	5.2047	14.9	2.623	0.081
		Fast High - Slow Low	15.24	6.0099	14.9	2.536	0.095
		Slow High - Fast Low	6.50	5.2047	14.9	1.249	0.607
		Slow High - Slow Low	8.09	6.0099	14.9	1.347	0.550
		Fast Low - Slow Low	1.59	6.0099	14.9	0.265	0.993
Fast first	RD	Fast High - Slow High	29.17	5.2047	14.9	5.605	**<0.001**
		Fast High - Fast Low	25.37	5.2047	14.9	4.874	**0.001**
		Fast High - Slow Low	29.67	6.0099	14.9	4.937	**0.001**
		Slow High - Fast Low	-3.80	5.2047	14.9	-0.730	0.884
		Slow High - Slow Low	0.50	6.0099	14.9	0.084	1.000
		Fast Low - Slow Low	4.30	6.0099	14.9	0.716	0.890
Together-late	RD	Fast High - Slow High	19.63	5.2047	14.9	3.772	**0.009**
		Fast High - Fast Low	22.48	5.2047	14.9	4.319	**0.003**
		Fast High - Slow Low	26.72	6.0099	14.9	4.447	**0.002**
		Slow High - Fast Low	2.85	5.2047	14.9	0.548	0.946
		Slow High - Slow Low	7.09	6.0099	14.9	1.180	0.648
		Fast Low - Slow Low	4.24	6.0099	14.9	0.706	0.893
Together-late	RD	Fast High - Slow High	14.78	5.2047	14.9	2.840	0.054
		Fast High - Fast Low	15.19	5.2047	14.9	2.918	**0.047**
		Fast High - Slow Low	17.84	6.0099	14.9	2.969	**0.043**
		Slow High - Fast Low	0.41	5.2047	14.9	0.079	1.000
		Slow High - Slow Low	3.06	6.0099	14.9	0.510	0.956

Continued on next page

Order	Site	Contrast	Estimate	SE	df	t-ratio	p-value
		Fast Low - Slow Low	2.65	6.0099	14.9	0.441	0.970
Slow first	RD	Fast High - Slow High	-9.70	5.2047	14.9	-1.864	0.284
		Fast High - Fast Low	9.76	5.2047	14.9	1.875	0.280
		Fast High - Slow Low	8.73	6.0099	14.9	1.453	0.488
		Slow High - Fast Low	19.46	5.2047	14.9	3.739	**0.010**
		Slow High - Slow Low	18.43	6.0099	14.9	3.067	**0.036**
		Fast Low - Slow Low	-1.03	6.0099	14.9	-0.171	0.998

S8 Priority effects

S8.1 The effect of arriving early

Table S13: Type III Analysis of Variance Table with Satterthwaite's Method on the full linear mixed effects model, including effect size of arriving early (ES_{early}) as a response variable, site (two levels: Lönnstorp and Röbacksdalen), arrival order (Order, two levels; fast-first and slow-first), sowing density (Density, two levels: high and low) and year (four levels; 2021, 2022, 2023 and 2024) as fixed effects, speciesID as random effects and block/site as nested random effect. Relevant comparisons are made using TukeyHSD.

Variable	SumSq	MeanSq	NumDF	DenDF	F-Value	P-Value	Tukey
Site (S)	1.4854	1.4854	1	450	7.7410	**0.006**	
Order (O)	9.4522	9.4522	1	450	49.2597	<**0.001**	
Density (D)	0.0198	0.0198	1	450	0.1032	0.748	
Year (Y)	9.4367	3.1456	3	450	16.3929	<**0.001**	
Site x Order	4.3778	4.3778	1	450	22.8148	<**0.001**	S14
Site x Density	0.2436	0.2436	1	450	1.2696	0.260	
Order x Density	0.0628	0.0628	1	450	0.3275	0.567	
Site x Year	1.0602	0.3534	3	450	1.8418	0.139	
Order x Year	11.2757	3.7586	3	450	19.5876	<**0.001**	
Density x Year	1.1988	0.3996	3	450	2.0825	0.102	
S x O x D	0.1647	0.1647	1	450	0.8582	0.355	
S x O x Y	2.1274	0.7091	3	450	3.6957	**0.012**	S15
S x D x Y	0.5824	0.1941	3	450	1.0117	0.387	
O x D x Y	1.2939	0.4313	3	450	2.2478	0.082	
S x O x D x Y	3.0583	1.0194	3	450	5.3127	**0.001**	S16

Table S14: TukeyHSD pairwise comparisons for the effect of arrival order and site (Röbäcksdalen (RD) and Lönnstorp (LT)) on ES_{early}, averaged over density and year levels.

**Contrast**	**Estimate**	**SE**	**df**	**t.ratio**	**p.value**
(Fast-first LT) - (Slow-first LT)	-0.1082	0.0749	444.14	-1.445	0.472
(Fast-first LT) - (Fast-first RD)	0.0962	0.0583	17.59	1.651	0.378
(Fast-first LT) - (Slow-first RD)	-0.4730	0.0656	27.86	-7.208	<**0.001**
(Slow-first LT) - (Fast-first RD)	0.2045	0.0710	36.97	2.881	0.032
(Slow-first LT) - (Slow-first RD)	-0.3648	0.0771	50.29	-4.732	**0.001**
(Fast-first RD) - (Slow-first RD)	-0.5693	0.0611	442.59	-9.323	<**0.001**

Table S15: TukeyHSD pairwise comparisons for the effect of arrival order, year, and site (Röbäcksdalen (RD) and Lönnstorp (LT)) on ES_{early}, averaged over density levels.

**Site**	**Order**	**Contrast**	**Estimate**	**SE**	**df**	**t.ratio**	**p.value**
LT	Fast-first	Year2021 - Year2022	-0.1098	0.1339	447.10	-0.820	0.845
		Year2021 - Year2023	-0.2190	0.1376	445.70	-1.592	0.384
		Year2021 - Year2024	-0.1215	0.1396	447.02	-0.870	0.820
		Year2022 - Year2023	-0.1092	0.1114	442.55	-0.980	0.761
		Year2022 - Year2024	-0.0117	0.1138	442.63	-0.103	1.000
		Year2023 - Year2024	0.0975	0.1182	442.74	0.825	0.843
RD	Fast-first	Year2021 - Year2022	0.3494	0.1017	444.93	3.434	**0.004**
		Year2021 - Year2023	0.3797	0.1006	445.24	3.773	**0.001**
		Year2021 - Year2024	0.1461	0.1048	445.46	1.394	0.504
		Year2022 - Year2023	0.0303	0.1078	442.74	0.281	0.992
		Year2022 - Year2024	-0.2033	0.1117	442.94	-1.821	0.265
		Year2023 - Year2024	-0.2336	0.1106	443.02	-2.111	0.151
LT	Slow-first	Year2021 - Year2022	-0.5700	0.1719	443.48	-3.316	**0.005**
		Year2021 - Year2023	-0.7804	0.1942	446.50	-4.018	**0.001**
		Year2021 - Year2024	-1.0858	0.1692	448.13	-6.417	<**0.001**
		Year2022 - Year2023	-0.2104	0.1715	445.11	-1.226	0.611
		Year2022 - Year2024	-0.5158	0.1424	445.99	-3.621	**0.002**
		Year2023 - Year2024	-0.3054	0.1680	443.70	-1.818	0.266
RD	Slow-first	Year2021 - Year2022	-0.7485	0.1321	442.55	-5.669	<**0.0001**
		Year2021 - Year2023	-0.8058	0.1234	444.85	-6.531	<**0.001**
		Year2021 - Year2024	-0.8032	0.1279	444.22	-6.281	<**0.001**
		Year2022 - Year2023	-0.0572	0.1442	443.14	-0.397	0.979
		Year2022 - Year2024	-0.0547	0.1481	443.00	-0.369	0.983
		Year2023 - Year2024	0.0026	0.1404	444.80	0.018	1.000

Table S16: TukeyHSD pairwise comparisons for the effect of site (Röbäcksdalen (RD) and Lönnstorp (LT)), arrival order, year and density on ES_{early}.

Site	Order	Year	Contrast	Estimate	SE	df	t.ratio	p.value
LT	Fast-first	2021	High - Low	0.2575	0.2209	447.22	1.165	0.245
		2022	High - Low	-0.0994	0.1510	443.75	-0.658	0.511
		2023	High - Low	-0.2978	0.1639	442.14	-1.817	0.070
		2024	High - Low	-0.0842	0.1704	443.28	-0.494	0.621
RD	Fast-first	2021	High - Low	-0.1902	0.1327	443.51	-1.433	0.153
		2022	High - Low	0.1293	0.1539	443.32	0.840	0.401
		2023	High - Low	0.4510	0.1510	443.75	2.987	**0.003**
		2024	High - Low	0.1787	0.1618	442.68	1.104	0.270
LT	Slow-first	2021	High - Low	0.0310	0.2755	446.66	0.113	0.910
		2022	High - Low	0.2543	0.2069	444.89	1.229	0.220
		2023	High - Low	0.1750	0.2744	448.05	0.638	0.524
		2024	High - Low	-0.5475	0.1961	448.57	-2.791	**0.006**
RD	Slow-first	2021	High - Low	0.5804	0.1539	443.32	3.770	<**0.001**
		2022	High - Low	-0.2245	0.2146	442.14	-1.046	0.296
		2023	High - Low	-0.1554	0.1926	444.29	-0.807	0.420
		2024	High - Low	-0.2103	0.2041	443.90	-1.030	0.303

S8.2 The effect of arriving late

Table S17: Type III Analysis of Variance Table with Satterthwaite's Method on the full linear mixed effects model, including effect size of arriving late as a response variable, site (two levels: Lönnstorp and Röbacksdalen), arrival order (Order, two levels; fast-first and slow-first), sowing density (Density, two levels: high and low) and year (four levels; 2021, 2022, 2023 and 2024) as fixed effects, species and year as random effects and block/site as nested random effect. Relevant comparisons are made using TukeyHSD.

Variable	SumSq	MeanSq	NumDF	DenDF	F-Value	P-Value	Tukey
Site (S)	6.8072	6.8072	1	381.72	30.16	<**0.001**	
Order (O)	0.9363	0.9363	1	9.30	4.15	0.071	
Density (D)	0.3997	0.3997	1	9.30	1.77	0.215	
Year (Y)	14.3910	4.7970	3	349.32	21.26	<**0.001**	
Site x Order	4.1656	4.1656	1	381.51	18.46	<**0.001**	S18
Site x Density	0.0006	0.0006	1	381.29	0.003	0.959	
Order x Density	0.5689	0.5689	1	9.31	2.52	0.146	
Site x Year	5.0633	1.6878	3	378.41	7.48	<**0.001**	S19
Order x Year	4.9241	1.6414	3	350.02	7.27	<**0.001**	
Density x Year	3.3808	1.1269	3	350.13	4.99	**0.002**	
S x O x D	0.1881	0.1881	1	380.62	0.83	0.362	
S x O x Y	0.4410	0.1470	3	378.09	0.65	0.582	
S x D x Y	0.5280	0.1760	3	378.25	0.78	0.506	
O x D x Y	3.8100	1.2700	3	350.64	5.63	**0.001**	S20
S x O x D x Y	0.3560	0.1187	3	378.15	0.53	0.665	

Table S18: TukeyHSD pairwise comparisons for the effect of arrival order and site (Röbäcksdalen (RD) and Lönnstorp (LT)) on ES_{late}, averaged over density and year levels.

**Contrast**	**Estimate**	**SE**	**df**	**t.ratio**	**p.value**
(Fast-first LT) - (Slow-first LT)	0.0372	0.1238	24.80	0.300	0.990
(Fast-first LT) - (Fast-first RD)	0.5702	0.0979	56.62	5.823	<**0.001**
(Fast-first LT) - (Slow-first RD)	0.1069	0.1209	20.86	0.884	0.813
(Slow-first LT) - (Fast-first RD)	0.5331	0.1203	19.63	4.430	**0.001**
(Slow-first LT) - (Slow-first RD)	0.0697	0.0653	14.29	1.067	0.714
(Fast-first RD) - (Slow-first RD)	-0.4634	0.1171	21.34	-3.957	**0.004**

Table S19: Tukey HSD pairwise comparisons by year for each site (Röbäcksdalen (RD) and Lönnstorp (LT)). Results are averaged over the levels of Order and Density.

**Site**	**Contrast**	**Estimate**	**SE**	**df**	**t.ratio**	**p.value**
LT	Year2021 - Year2022	0.2352	0.1280	378.11	1.837	0.258
	Year2021 - Year2023	0.2503	0.1368	369.86	1.830	0.261
	Year2021 - Year2024	0.2233	0.1227	378.81	1.820	0.266
	Year2022 - Year2023	0.0152	0.1239	377.51	0.122	0.100
	Year2022 - Year2024	-0.0119	0.1133	380.63	-0.105	0.100
	Year2023 - Year2024	-0.0270	0.1182	379.73	-0.229	0.100
RD	Year2021 - Year2022	0.7395	0.1037	383.41	7.128	<**0.001**
	Year2021 - Year2023	0.9114	0.1093	383.32	8.336	<**0.001**
	Year2021 - Year2024	0.9441	0.1144	379.23	8.256	<**0.001**
	Year2022 - Year2023	0.1720	0.1137	371.77	1.513	0.431
	Year2022 - Year2024	0.2046	0.1191	376.85	1.718	0.316
	Year2023 - Year2024	0.0326	0.1188	371.82	0.275	0.993

Table S20: Pairwise comparisons for sowing density (High vs. Low) across different combinations of Order and Year. Results are averaged over the levels of Site.

**Order**	**Year**	**Contrast**	**Estimate**	**SE**	**df**	**t.ratio**	**p.value**
Fast-first	2021	High - Low	-1.0842	0.2300	51.74	-4.714	<**0.001**
Fast-first	2022	High - Low	-0.0953	0.2357	49.63	-0.404	0.688
Fast-first	2023	High - Low	-0.2082	0.2485	64.49	-0.838	0.405
Fast-first	2024	High - Low	0.1657	0.2426	60.17	0.683	0.497
Slow-first	2021	High - Low	0.0677	0.1900	47.10	0.356	0.723
Slow-first	2022	High - Low	-0.0945	0.1626	27.04	-0.581	0.566
Slow-first	2023	High - Low	0.0409	0.1766	31.07	0.232	0.818
Slow-first	2024	High - Low	0.0936	0.1719	29.29	0.545	0.590
